# Quantum Transport of Particles and Entropy

**DOI:** 10.3390/e23121573

**Published:** 2021-11-25

**Authors:** Christoph Strunk

**Affiliations:** Institute of Experimental and Applied Physics, University of Regensburg, D-93025 Regensburg, Germany; christoph.strunk@ur.de

**Keywords:** thermodynamics, quantum transport, thermoelectricity, transport equations

## Abstract

A unified view on macroscopic thermodynamics and quantum transport is presented. Thermodynamic processes with an exchange of energy between two systems necessarily involve the flow of other *balancable* quantities. These flows are first analyzed using a simple drift-diffusion model, which includes the thermoelectric effects, and connects the various transport coefficients to certain thermodynamic susceptibilities and a diffusion coefficient. In the second part of the paper, the connection between macroscopic thermodynamics and quantum statistics is discussed. It is proposed to employ not particles, but *elementary Fermi- or Bose-systems* as the elementary building blocks of ideal quantum gases. In this way, the transport not only of particles but also of entropy can be derived in a concise way, and is illustrated both for ballistic quantum wires, and for diffusive conductors. In particular, the quantum interference of entropy flow is in close correspondence to that of electric current.

## 1. Introduction

The transport of physical quantities such as energy, momentum, angular momentum, electrical charge ($E,\vec{P},\vec{L},Q$) can be described in terms of the flow of suitably chosen particles or quasiparticles, which carry the other physical quantities in the sense that with each particle a certain amount of $E,\vec{P},\vec{L},Q$ is associated, which flows along with the particles. Although this statement seems very intuitive, it breaks down in the context of thermal transport or ‘heat’ conduction. One reason is that ‘heat’ is not a state quantity, and thus cannot be associated as ‘heat content’ with a volume element, or a certain number of particles.

During the development of thermodynamics, it had become apparent that the traditional concept of heat had to be split into two more abstract concepts [1]: one, *entropy*, which is specific for thermal phenomena, and a second one, *energy*, which is relevant in all branches of physics. The terms ‘heat’ and ‘heat current’ survived in modern physics as a concept characteristic for *processes*, namely the amount of energy transferred from one system into another together with a given amount of entropy. This notion of heat, however, is problematic: even though heat cannot be balanced [2], nor linked to quantum states [3] it often competes with the much more powerful concept of entropy. Even today, entropy is less popular than heat [4], possibly because of persistent tradition, and its incompatibility with classical mechanics [5].

On the other hand, entropy can be handled very easily at the macroscopic level, as it behaves as an analogue of electric charge (except for the property of conservation). In this review, we take the notion of entropy as the starting point for thermodynamics—in the same way as one takes the notion of electric charge as the starting point for electricity. We do not ask what electric charge or entropy actually ‘are’, but take them as fundamental concepts, which prove useful in the quantitative description of electric or thermal phenomena, respectively. An intuition for both charge and entropy can be developed only via the many examples, where we see them at work. To avoid the conceptional difficulties of ‘heat’, one often speaks of energy transport when heat transport is meant. Such language, however, obscures that there are usually also other—non-thermal, but often equally important—contributions to the energy transport. This is most obvious in the context of thermoelectric phenomena, which are a central topic of this review. It is semantically and conceptually more appropriate to ascribe the thermal transport to the simultaneous flow of energy *and* entropy. A description of transport in terms of the motion of particles can be very easily visualized. However, it is hard to avoid the traps of classical physics in doing so. The reason is that quantum properties, in particular the indistinguishability of identical particles, have no counterpart in the classical world, although they clearly show up in the thermodynamic and transport properties of matter at the macroscopic level.

Our goal is a self-contained description of transport processes in systems of indistinguishable particles, which does *not* contain elements incompatible with the statistical concepts of quantum physics. We will elucidate the relation between the flows of particles and entropy. We will see that as opposed to many other quantities, the entropy cannot be viewed as ‘carried by particles’. In contrast, energy, particles and entropy (other quantities involved) are carried in an identical way by different entities that we propose to label *elementary Fermi-* or *Bose-systems*. The terms Bose- and Fermi- refer to the indistinguishability of (quasi)-particles, and reflect the fact that entropy is always of a quantum nature, and can never fully be understood in classical terms.

The review is organized as follows: in Section 2 we first formulate thermodynamics in a compact way that is appropriate for the investigation of transport processes. In Section 3 we present the simplest of all transport theories, the drift-diffusion model, which has the great advantage of providing a simple intuitive picture of diffusive transport. In this approach particles and entropy are treated on the same footing. In Section 4 we introduce *elementary Fermi-and Bose-systems* as the elementary building blocks of quantum gases, and derive their thermodynamic equations of state (EoS). In Section 6 and Section 7 we exploit these EoS to formulate ballistic transport in one-dimensional quantum wires in the Landauer-Büttiker approach. In Section 9 we show that the very same equations of state can be applied to generalize the drift-diffusion model in a way, which is equivalent to the Boltzmann equation in relaxation-time approximation. From this perspective, there is no fundamental difference between classical and quantum transport. In Section 10 some implications of our approach are discussed—in particular, it is shown that also quantum interference can be included into the discussion of thermal transport phenomena.

## 2. Thermodynamics

Thermodynamics can be based on the postulate that the static properties of any physical system with *r* independent variables can be compressed into certain functions of these variables, which are called *thermodynamic potentials* [6,7,8]. The most familiar thermodynamic potential is the energy *E* when expressed as a function of the independent *extensive* variables of the system. In the case of simple fluid or gas, the independent extensive variables are the entropy *S*, the volume *V* and the particle number *N*. Assigning numerical values for a set of independent variables, e.g., for {S,V,N}, specifies a certain *state* of the system. The total differential of the function E(S,V,N) takes the form

(1) dE=TdS−pdV+μdN,

where the intensive quantities absolute temperature *T*, (negative) pressure *p*, and chemical potential μ are defined as the partial derivatives of E(S,V,N) with respect to *S*, *V*, and *N*.

Depending on the specific problem under consideration, it is often convenient to use the method of Legendre transform to exchange any of the extensive variables {S,V,N} with its intensive partner. If, e.g., {T,V,N} are chosen as set of independent variables the corresponding thermodynamic potential is the free energy F(T,V,N)=E(T,V,N)−T·S(T,V,N). As we will see below, the grand-canonical, or Landau-potential

(2) K(T,V,μ)=E−TS−μN

is best adapted for the transport processes (in particular in solids), as it refers to {T,V,μ} as set of independent variables.

The final element of thermodynamics needed for the present work is the homogeneity postulate. Homogeneity of E(S,V,N) means [9] that *E* can be written as

(3) E(S,V,N)=V·e(s,n),

where we define e=E/V as the energy density, s=S/V as the entropy density, and as n=N/V the particle density. Equation (3) also implies the Euler or homogeneity relation

(4) E=TS−pV+μN.

The physical meaning of the homogeneity is quite fundamental: it means that systems obey a scaling relation, which allows one to formulate the relations between their state variables in a way that is independent of the ‘size’ of the system. Long-ranged interactions can be included into thermodynamics via the gravitational or electrostatic potential (see Equation (13) below [10]). It follows that all properties of a system can be expressed by relations between the local densities e,s and, *n*, as well as the *intensive* quantities T,p, and μ. The function e(s,n) represents a *reduced* thermodynamic potential, which still contains all thermodynamic information about the system, except its volume, and constitutes a *local* formulation of thermodynamics, which is well suited for the description of spatially inhomogeneous situations, in which transport phenomena can occur. The reduced Gibbs fundamental form corresponding to e(s,n) reads

(5) de=Tds+μdn.

If we choose e(s,n) as reduced thermodynamic potential, we can still apply the formalism of Legendre transforms to exchange the independent variables, i.e., *s* with *T* and *n* with μ. If we do so, we find using Equation (4)

(6) −p(T,μ)=e(T,μ)−T·s(T,μ)−μ·n(T,μ)

as the corresponding reduced thermodynamic potential. The differential of p(T,μ) represents a *reduced* fundamental form, which is also known as the Gibbs–Duhem relation

(7) dp=sdT+ndμ,

with the two equations of state

(8) $$s\left(T,\mu \right)\;=\;\frac{\partial p(T,\mu )}{\partial T}\;\mathrm{and}\;n\left(T,\mu \right)\;=\;\frac{\partial p(T,\mu )}{\partial \mu }\;.$$

If the pressure of a function of *T* and μ is well behaved (i.e., twice continuously differentiable), mathematics tells us that

(9) $$\frac{\partial s(T,\mu )}{\partial \mu }\;=\;\frac{\partial^{2}p\left(T,\mu \right)}{\partial \mu \;\partial T}\;=\;\frac{\partial^{2}p\left(T,\mu \right)}{\partial T\;\partial \mu }\;=\;\frac{\partial n(T,\mu )}{\partial T}\;.$$

Relations of this type are called Maxwell’s relations [11] and we will exploit them below for an elementary derivation of Onsager reciprocity.

Using Equation (4), we see that pressure p(T,μ) is equivalent to the Landau-potential

(10) K(T,V,μ)=−Vp(T,μ).

It is appealing that also conservative force fields such as the gravitational field or the electrostatic field can be built into the local thermodynamics. In a system with electrically charged particles we have one more extensive variable, i.e., the electric charge *Q*, which provides an extra term in the Gibbs fundamental form (see Equation (1)):

(11) dE=TdS−pdV+μdN+ϕdQ.

The to *Q* thermodynamically conjugate variable is the electrostatic potential ϕ, which determines the electrostatic contribution to the energy required for a local increase of the charge density.

In many important examples charge and particle number are connected by a characteristic constant of the system, i.e., the charge per particle $\hat{q}$ [12]. In these cases, charge and particle number are not independent, but proportional: $Q=\hat{q}N$, implying that we can combine the last two terms in Equation (11) into one:

(12) $$dE\;=\;T\;dS-p\;dV+\bar{\mu }\;dN\;$$

where

(13) $$\bar{\mu }\;:=\;\mu +\hat{q}\phi$$

is called the *electrochemical potential*. For charged particles it is $\bar{\mu }$ and not μ, which quantifies the energy changes required for adding or removing particles. Hence $\bar{\mu }$ and not just μ enters all thermodynamic relations for systems of charged particles [13].

Importantly, only those physical quantities can be transported for which a *balance* is possible, which tells us which amount of this quantity has left at time $t_{1}$ a certain volume element $V_{1}$ in space and has arrived at a time $t_{2}$ in another volume element $V_{2}$ in space. The prototype of such balances are those of *amounts* of a substance. It is suggested to call the physical quantities allowing analogous operations *balancable* or *substance-like*.

Please note that balancable quantities are not necessarily conserved: important examples are entropy, particle number, or spin. Total angular momentum is of course conserved, but the spin of the moving particles under consideration may be transferred to other systems, i.e., by spin-dependent scattering processes. Entropy can be generated, without extracting it from another system. Particles such as photons, phonons or excitons can be created or annihilated, provided that the canonic conservation laws for energy, electric charge, momentum, and angular momentum are obeyed.

So far we have not exploited any of the conservation laws. In general, a balancable quantity *X* must obey a *continuity equation*

(14) $$\frac{\partial x(t,\vec{r})}{\partial t}+\nabla {\vec{j}}_{X}\left(t,\vec{r}\right)\;=\;\Sigma_{X}\left(t,\vec{r}\right)\;,$$

where *x* is the local *X*-density, ${\vec{j}}_{X}$ the *X*-current density and $\Sigma_{X}$ the *X*-generation rate per volume. For conserved quantities $\Sigma_{X}$ vanishes.

Using the reduced GFF (Equation (5)), we can write the time derivative of energy density

(15) $$\frac{\partial e\left[s\left(t,\vec{r}\right),n\left(t,\vec{r}\right)\right]}{\partial t}\;=\;T\;\frac{\partial s}{\partial t}+\bar{\mu }\;\frac{\partial n}{\partial t}\;.$$

Assuming that *N* is conserved, the time derivatives of *e*, *s*, and *n* can be replaced by the negative divergences of the current densities, $-\nabla {\vec{j}}_{E}$ and $-\nabla {\vec{j}}_{N}$, and $\Sigma_{S}-\nabla {\vec{j}}_{S}$, respectively,

(16) $$\nabla {\vec{j}}_{E}\;=\;T\;\left(\nabla {\vec{j}}_{S}\;-\;\Sigma_{S}\right)\;+\;\bar{\mu }\;\nabla {\vec{j}}_{N}\;.$$

**Figure 1:**
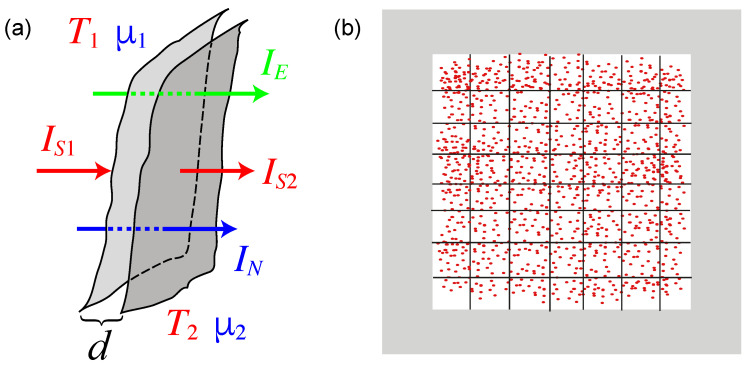
(**a**) An energy current $I_{E}$ carried by and entropy current $I_{S}$ and a particle current $I_{N}$ current flows through two isothermal surface elements with constant temperatures $T_{1}$ and $T_{2}≲T_{1}$ and electrochemical potentials ${\bar{\mu }}_{1}$ and ${\bar{\mu }}_{2}≲{\bar{\mu }}_{1}$. Although $I_{E}$ and $I_{N}$ are constant, $I_{S}$ is not. The rate of entropy production between the two surfaces becomes negligible compared to the entropy currents through the surface elements, as the distance *d* and the differences $T_{1}-T_{2}$ and ${\bar{\mu }}_{1}-{\bar{\mu }}_{2}$ go to zero. (**b**) A container with a gas of (quasi)-particles can be decomposed into small subvolumina with almost arbitrary size. Each subvolume represents another realization of the system ‘gas’, which continuously exchanges energy, entropy and particles with its neighbors. In the presence of a gradient of *T* or μ a subvolume can be considered to be in local equilibrium, provided that its size is about $\Lambda^{3}$.

Applying Gauss’ theorem to a slice-shaped volume element with small thickness *d* and volume ΔV (see Figure 1a), we obtain

(17) $$-\frac{T_{1}+T_{2}}{2}\cdot \Sigma_{S}\cdot \Delta V\;=\;T_{1}I_{S,1}-T_{2}I_{S,2}\;=\;I_{E}-\Delta \bar{\mu }\cdot I_{N}\;.$$

Although $I_{E}$ and $I_{N}$ are constant within the slice, the entropy current increases from one surface of the slice to the other by $\Sigma_{S}\cdot \Delta V$. If the thickness of the slice (and thus ΔV) goes to zero, the entropy production rate $\Sigma_{S}\cdot \Delta V$ becomes negligible against the average $I_{S}=\left(I_{S,1}+I_{S,2}\right)/2$. On a surface of constant *T* and $\bar{\mu }$ we thus obtain:

(18) $$I_{E}\;=\;TI_{S}+\bar{\mu }\;I_{N}\;.$$

This expression tells the total strength of the energy current that is ‘carried’ by the entropy and particle currents, namely $T\;I_{S}$, and $\bar{\mu }\;I_{N}$, respectively [14]. The term $T\;I_{S}$ in this relation is usually called the ‘heat current’. However, as we have stressed already, ‘heat’ is not a state variable, to which one can assign a value in a specific state of a system. This has led to the peculiar notion of heat as “energy in transit”, i.e., heat being the strange thing that is flowing only, but disappears upon arrival in any system.

## 3. Drift-Diffusion Model

**Figure 2:**
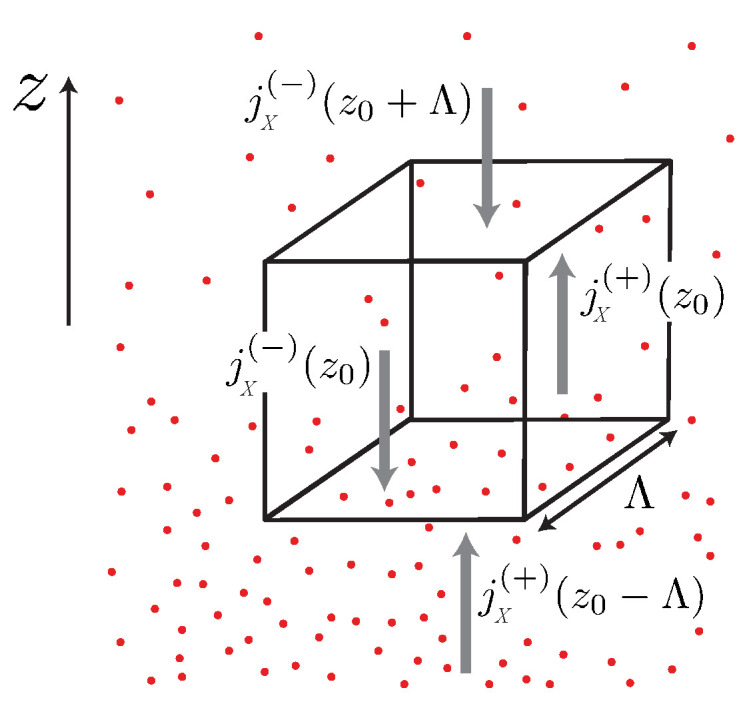
Elementary derivation of the diffusion constant in three dimensions: summing up the four contributions $j_{Xz}=\pm \frac{1}{6}\;x\left(z\right)\cdot \langle |\vec{v}|\rangle$ to the *z*-component $j_{Xz}$ of the *X*-current density through the top and bottom surface of a cube of dimension Λ, one arrives in linear approximation at Equation (20), if the *z*-direction is chosen parallel to the gradient $\nabla x(t,\vec{r})$ of the *X*-density.

From the point of view of macroscopic thermodynamics, it is natural to describe non-equilibrium situations by spatially varying thermodynamic variables, i.e., the local values of the densities and the intensive variables. To do so, we decompose a gas of a fluid into small subvolumina, which can still be considered to be in local equilibrium, as illustrated in Figure 1b. An obvious question is, what is the length scale, below which the concepts of a local temperature and a local chemical potential are no longer applicable? It is natural to identify this length scale with the mean free path Λ of the scattering processes, which are responsible for establishing local equilibrium. For smaller distances it is not possible to assign a *T*- or μ-difference. In the simplest case, the mean free path is given by

(19) $$\Lambda \;=\;\frac{1}{n_{\mathrm{scatt}}\;\sigma_{c}}\;,$$

where $n_{\mathrm{scatt}}$ is the density of scatterers, and $\sigma_{c}$ is the cross section of the relevant scattering process [15]. If the mobile particles in the system move with average velocity $\langle |\vec{v}|\rangle$ between the scattering events [16]. Λ can be translated into a scattering time τ via the relation $\Lambda =\langle |\vec{v}|\rangle \cdot \tau$. The elementary consideration illustrated in Figure 2 shows that the current density ${\vec{j}}_{X}$ associated with a balancable quantity *X* can be written in linear approximation as

(20) $${\vec{j}}_{X}\;=\;-D\cdot \nabla x\left(t,\vec{r}\right)\;,$$

where

(21) $$D\;=\;\frac{1}{3}\langle |\vec{v}|\rangle \Lambda$$

is the diffusion constant [17,18].

At face value this derivation appears to be based very much on classical physics. Looking more closely, however, the only thing really exploited it the condition that the quantity *X* is *balancable*, i.e., that it is possible to say what is the net amount of *X* transported in the direction of the gradient $\nabla x(t,\vec{r})$ of the *X*-density. For this reason, the model is extremely robust and holds in both classical and quantum physics. Moreover, on thermodynamics, it relies on only two additional concepts, i.e., the mean transport velocity $\langle |\vec{v}|\rangle$ and the mean free path Λ between scattering events. In quantum physics,

$$\vec{v}\left(\vec{k}\right)\;=\;\frac{\partial \varepsilon (\vec{k})}{\hbar \partial \vec{k}}$$

is the group velocity resulting from the dispersion relation $\varepsilon (\vec{k})$ of the matter waves with wave vector $\vec{k}$, while Λ is derived from the quantum-mechanical scattering cross section $\sigma_{c}$ (see Equation (19)).

To deal with particle currents, we set X=N, and obtain from Equation (20)

(22) $${\vec{j}}_{N}\;=\;-D\cdot \nabla n\left(t,\vec{r}\right)\;,$$

which is known as Fick’s 1. law.

Applying Equation (20) to the electric charge, we must choose *T* and $\bar{\mu }$ (Equation (13)) as independent variables. In addition, we must take into account that even for $\nabla n(t,\vec{r})=0$, a so-called drift current can be present, which is driven by the electric field. Adding this term to the diffusion current (Equation (22)), we obtain for the electric current

(23) $$\begin{array}{ccc} {\vec{j}}_{Q}\; & = & \;-\hat{q}D\cdot \nabla n\left(t,\vec{r}\right)-\sigma \nabla \phi \left(t,\vec{r}\right)\\ & = & \;-\hat{q}D\frac{\partial n(T,\mu )}{\partial \mu }\;\nabla \mu -\sigma \;\nabla \phi -\hat{q}D\frac{\partial n(T,\mu )}{\partial T}\;\nabla T\;, \end{array}$$

where σ is the electric conductivity. In global equilibrium *T* and $\bar{\mu }=\mu \left(\vec{r}\right)+\hat{q}\phi \left(\vec{r}\right)$ are spatially constant. In this case, the charge current must vanish and one obtains the *Einstein relation* between *D* and σ:

(24) $$\sigma \;=\;{\hat{q}}^{2}\frac{\partial n(T,\mu )}{\partial \mu }\cdot D\;.$$

Please note that in equilibrium the gradient of $\bar{\mu }$ must vanish, but not those of μ and ϕ separately, a phenomenon, which occurs, e.g., in the vicinity of a pn-junction in inhomogeneous semiconductors.

The thermodynamic susceptibility

(25) $$\nu \;=\;\frac{\partial n(T,\mu )}{\partial \mu }\;=\;n^{2}\kappa_{T}$$

is closely related to the isothermal compressibility $\kappa_{T}=-\left(1/\hat{v}\right)\cdot \partial \hat{v}\left(T,p\right)/\partial p$. Here, we propose to call ν the *particle capacity*, as it tells us how many particles can be added to the system, if the (electro)-chemical potential is raised by a certain amount [19].

In systems of charged particles, particle capacity is related to the electric capacitance. In low-dimensional conductors ∂n(T,μ)/∂μ can be very small and then provides a contribution to the total inverse capacitance, which cannot be neglected against that of the geometrical (electrostatic) capacitance. The sum of both contributions determines the ratio between the electric charge on a capacitor and the electrochemical potential difference between its electrodes. In the context of low-dimensional conductors ν is also called ‘quantum capacitance’. This term was introduced in the context of two-dimensional electron systems [20]. Graphene is a another fashionable example, where ∂n(T,μ)/∂μ approaches zero at the Dirac-point [21].

With the Einstein relation we can rewrite Equation (23) in the more compact form

(26) $${\vec{j}}_{Q}\;=\;-\frac{\sigma }{\hat{q}}\cdot \nabla \bar{\mu }\left(t,\vec{r}\right)-\hat{q}D\frac{\partial n(T,\mu )}{\partial T}\;\nabla T\;.$$

We see that a charge current can not only be driven by a gradient of the electrochemical potential, but also by a temperature gradient. The second term in Equation (26) describes thermoelectricity, i.e., the *Seebeck effect*. To identify the prefactor in front of ∇T we employ Equation (9) and find:

(27) $$\frac{\partial n(T,\mu )}{\partial T}\;=\;\frac{\partial s(T,\mu )}{\partial \mu }\;=\;\frac{\partial s(T,n)}{\partial n}\cdot \frac{\partial n(T,\mu )}{\partial \mu }\;.$$

**Figure 3:**
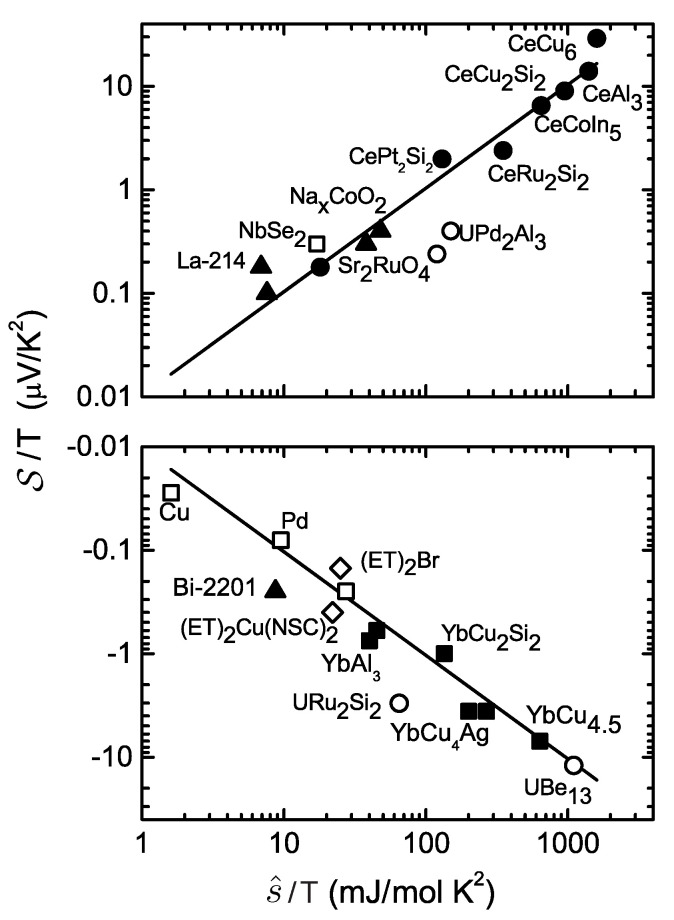
Thermopower over temperature S/T versus molar entropy over temperature $\hat{s}\left(T\right)/T$ in the limit T→0 for many different metallic compounds. For electron-like $\left(\hat{q}<0\right)$ conduction S is negative (lower panel), while for hole-like $\left(\hat{q}>0\right)$ conduction S is positive (upper panel); Solid circles (squares) represent Ce (Yb) heavy-fermion systems. Uranium-based compounds are represented by open circles, metallic oxides by solid triangles, organic conductors by open diamonds, and common metals by open squares. For some data points, due to the lack of space, the name of the compound is not explicitly mentioned. The two solid lines represent $\hat{s}/\left(\hat{q}T\right)$ and are motivated by the discussion leading to Equation (87) below (adapted from [22]).

Thus, we arrive at

$${\vec{j}}_{Q}\;=\;-\;\sigma \cdot \left\{\frac{\nabla \bar{\mu }\left(t,\vec{r}\right)}{\hat{q}}\;+\;S\cdot \nabla T\left(t,\vec{r}\right)\right\}\;,$$

where

(28) $$S\;=\;\frac{1}{\hat{q}}\;\frac{\partial s(T,n)}{\partial n}$$

is called Seebeck coefficient, or thermopower. The Seebeck coefficient is closely related to, but not precisely identical with the entropy per particle $\hat{s}$. This close correspondence has been experimentally verified for a broad range of metals with heavy electron-like and hole-like quasiparticles and is illustrated in Figure 3, where the molar entropy (which in this case is identical to the molar heat capacity; see Equation (49) below) and the thermopower at the lowest temperatures vary over three orders of magnitude, while their ratio remains close to $\hat{q}$ [22].

Next, let us consider the transport of entropy. If we choose *T* and *n* as independent variables, we can apply Equation (20) to entropy (i.e., we set X=S), and obtain the entropy current density, and the thermal contribution to the energy current density again as the sum of a diffusion and a drift term:

(29) $$\begin{array}{ccc} T\cdot {\vec{j}}_{S}\; & = & \;-T\cdot D\nabla s(T,n)\;-\;\Pi \sigma \nabla \phi \\ & = & \;-T\cdot D\left\{\frac{\partial s(T,n)}{\partial T}\nabla T+\frac{\partial s(T,n)}{\partial n}\nabla n\right\}-\Pi \sigma \nabla \phi \end{array}$$

(30) $$\begin{array}{ccc} & = & \;-Dc_{v}\nabla T+T\cdot \frac{\partial s(T,n)}{\partial n}\;{\vec{j}}_{N}\; \end{array}$$

where $c_{v}=T\partial s\left(T,n\right)/\partial T=\partial e\left(T,n\right)/\partial T$ is the heat capacity per volume at constant density n=N/V. The first term in Equation (29) represents Fourier’s law, i.e., the *conductive* thermal contribution to the energy current density defines the thermal conductivity

(31) $$\lambda =Dc_{v}=\frac{1}{3}n{\hat{c}}_{v}\langle |\vec{v}{|\rangle }^{2}\tau \;.$$

The second term in Equation (29) represents the drift contribution to the Peltier effect. At constant *T*, the entropy content of moving particles results in a *convective* thermal contribution to the energy current. The Peltier coefficient connects the convective part of the thermal current density $T{\vec{j}}_{S}$ and the electric current density ${\vec{j}}_{Q}=\hat{q}{\vec{j}}_{N}$. If we request that the diffusion and drift contributions to ${\vec{j}}_{N}$ contribute in the same way to ${\vec{j}}_{S}$, the Peltier coefficient must read:

(32) $$\Pi \;=\;\frac{T}{\hat{q}}\;\frac{\partial s(T,n)}{\partial n}\;.$$

From Equation (27), and the assumption that the diffusion coefficient *D* in Equation (20) characterizes both the entropy and the particle current density, it follows that S and Π obey the Kelvin-Onsager relation [23]

(33) Π=T·S.

Applying the general transport equation Equation (20) to the energy density e(T,μ) one can easily verify that the generalized drift-diffusion model is consistent with Equation (16), if one uses the homogeneity relation (Equations (4) and (8)).

The main advantage of the drift-diffusion model is its extreme simplicity and generality. It does not depend on the nature of the diffusing (quasi)-particles, and works equally well for classical particles, fermions, or bosons. It is also independent of the dispersion relation of the particles under consideration, e.g., electrons, phonons or photons. This means, it can be used for almost all phenomena related to the diffusive transport of quasiparticles occurring in condensed matter physics. Introducing a single phenomenological parameter, the diffusion constant *D*, it relates all transport coefficients to certain thermodynamic susceptibilities.

That single parameter *D* is also its main deficiency, because *D* usually depends on the energy of the diffusing particles. As we will see in Section 9, this deficiency can be quite easily cured, if particles with different kinetic energy ε are associated with different subsystems of the gas, with an energy-dependent diffusion constant D(ε). This additional dependence on energy produces, in many cases, only a prefactor of order unity. Hence, the drift-diffusion model works quite well for the qualitative consideration of electric, thermal, and thermoelectric transport phenomena.

The drift-diffusion model is easily extended to two species of particles, e.g., electrons and holes in semiconductors. Another topical example is the transport of spin-up and spin-down electrons in the context of spintronics [24,25] and spin-caloritronics [26,27,28,29].

## 4. Elementary Fermi- and Bose-Systems

An essential issue of statistical physics is the decomposition of many-body systems into simpler thermodynamic subsystems, allowing for the calculation of the Landau-potential

(34) $$\begin{array}{ccc} K(T,V,\bar{\mu })\; & = & \;-k_{B}T\ln Z\left(T,V,\bar{\mu }\right)\;. \end{array}$$

via the grand partition function

(35) $$\;Z\left(T,V,\bar{\mu }\right)\;=\;\sum_{i}\;\exp\left(-\frac{E_{i}\left(V\right)-\bar{\mu }N_{i}}{k_{B}T}\right)\;.$$

For non-interacting quantum particles in a rectangular potential box, the volume enters the energy eigenvalues $E_{i}\left(V\right)$ via boundary conditions for the allowed $\vec{k}$-vectors.

If we restrict ourselves to this simplest case, the corresponding Hamiltonian reads in the language of 2nd quantization

(36) $$H=\sum_{k}\;\varepsilon \left(k\right)\;a_{k}^{†}a_{k}\;,$$

where the index *k* represents a wave vector, and ℏk is the momentum of a particle. The function ε(k) is the dispersion relation of these particles and $a_{k}^{†},a_{k}$ their creation and annihilation operators, respectively. The number operator is simply $N_{k}=a_{k}^{†}a_{k}$. When spin is of interest, its quantum number σ can be simply added to the index *k*; otherwise, it gives rise to an additional factor of 2 in front of the sum.

This Ansatz is very general, because many interacting systems can be transformed, at least approximately, into a Hamiltonian given by Equation (36). In many cases, residual interactions between the particles can be taken into account as a renormalization of the function ε(k), and a finite lifetime of the quasiparticle states resulting from scattering processes. Hence, the Hamiltonian in Equation (36) is relevant for a very broad class of systems, i.e., all systems with wave-like excitations. Not only conventional gases, but also complex many-body systems such as the lattice excitations of solids, Fermi- and Luttinger liquids, as well as elementary excitations in superfluids, superconductors, and (anti-)ferromagnets. Most of the quasiparticles of condensed matter physics can be described by the Hamiltonian in Equation (36).

The structure of Equation (36) suggest decomposing an ideal gas of particles or quasiparticles into simpler subsystems. Each subsystem is represented by a single term of the sum in Equation (36), i.e.,

$$H_{k}=\varepsilon \left(k\right)\;a_{k}^{†}a_{k}\;.$$

We propose to call these subsystems *elementary Fermi- or Bose-systems* [8], as they cannot be further decomposed into simpler subsystems. In a rectangular potential well [30] the elementary Fermi- or Bose-systems share the same volume *V* that also determines the allowed k(V) and have the following properties:

(a) *Elementary Fermi-systems* have only two eigenstates of H and N with the eigenvalues $E_{i}\in \left\{0,\varepsilon \right\}$, and, respectively, $N_{i}\in \left\{0,1\right\}$.
  The grand partition function of this system reads according to Equation (35)
  (37) $$Z_{F}\left(T,\bar{\mu }\right)=1+\exp\left(-\frac{\varepsilon \left(k\right)-\bar{\mu }}{k_{B}T}\right)\;.$$
(b) *Elementary Bose-systems* have an infinite, but countable number of eigenstates of H and N with the eigenvalues $E_{i}\in \left\{0,\varepsilon ,2\varepsilon ,3\varepsilon ,\ldots \right\}$, and $N_{i}\in \left\{0,1,2,3,\ldots \right\}$.
  The grand partition function is a geometric series in this case and reads:
  (38) $$\;Z_{B}\left(T,\bar{\mu }\right)=\frac{1}{1-\exp\left(-\frac{\varepsilon \left(k\right)-\bar{\mu }}{k_{B}T}\right)}\;.$$

From the partition functions we obtain the average particle numbers by differentiating $Z_{k}\left(T,V,\bar{\mu }\right)$ with respect to $\bar{\mu }$:

(39) $$\begin{array}{ccc} N_{k}\left(T,\bar{\mu }\right)\; & = & \;\frac{1}{\exp\left(\frac{\varepsilon \left(k\right)-\bar{\mu }}{k_{B}T}\right)\pm 1}\;. \end{array}$$

The $N_{k}$ are the well-known Fermi- (upper sign) and Bose-functions (lower sign), respectively. In Fermi-systems $N_{k}$ varies continuously between 0 and 1, while in Bose-systems $N_{k}$ varies continuously between 0 and *∞*. For Bose-systems $\bar{\mu }$ must be always smaller than ε(k)—otherwise the particle number of the system diverges. This divergence of $N_{k}$ at $\varepsilon_{k}=\bar{\mu }$ is the origin of Bose–Einstein condensation. It is important to note that the $N_{k}$ are average values, around which the particle number of the system labeled {k} statistically fluctuates, as opposed to the occupation probability of a single-particle state |k〉 used in Boltzmann theory [31]. The average particle numbers $N_{k}$ are often called *distribution functions*, since they tell how the total number of particles is distributed over the different elementary subsystems.

**Figure 4:**
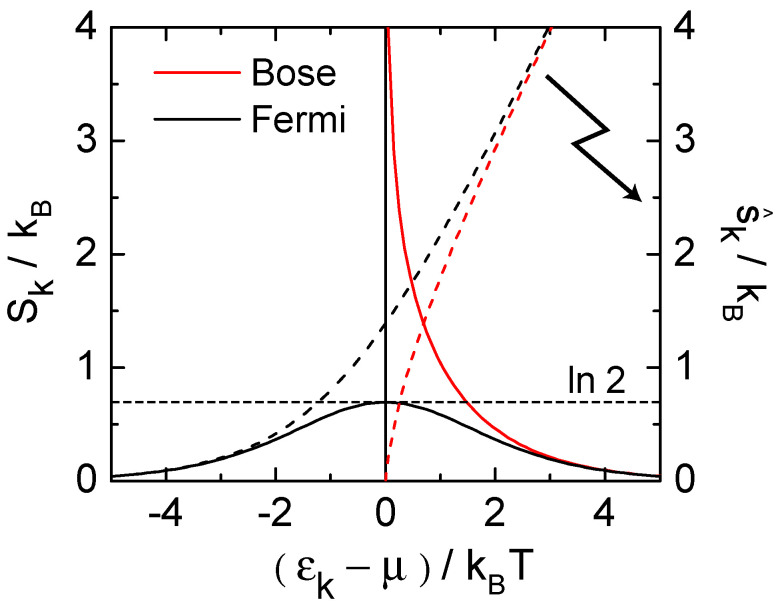
Entropy $S_{k}$ (solid lines) and entropy per particle ${\hat{s}}_{k}$ (dashed lines) of elementary Fermi- and Bose-systems with characteristic energy ε(k).

Using Equation (34), the Landau-potential of an elementary Fermi- and Bose-systems reads

(40) $$K_{k}\left(T,\bar{\mu }\right)=\;\mp \;k_{B}T\;\ln\left\{1\;\pm \;\exp\left(-\frac{\varepsilon \left(k\right)-\bar{\mu }}{k_{B}T}\right)\right\}\;,$$

where the upper sign holds for Fermi-, and the lower sign for Bose-systems, respectively. Next we compute the entropy of the elementary Fermi- and Bose-systems. Using Equations (2) and (34) one finds

(41) $$\begin{array}{ccc} S_{k}\left(T,\bar{\mu }\right) & = & -\frac{K_{k}-\left(\varepsilon \left(k\right)-\bar{\mu }\right)N_{k}}{T}\\ & = & \;\pm \;k_{B}\;\left\{\ln\left(1\pm \exp(-Y_{k})\right)\pm \;\frac{Y_{k}}{\exp(Y_{k})\pm 1}\right\}\;, \end{array}$$

with the abbreviation $Y_{k}=\left(\varepsilon \left(k\right)-\bar{\mu }\right)/k_{B}T$. The upper sign refers to Fermi- and the lower sign to Bose-systems, respectively. For high energies $\varepsilon \gg \bar{\mu }$ the second term in Equation (41) dominates, and the entropy per particle approaches

(42) $${\hat{s}}_{k}=\frac{\varepsilon \left(k\right)-\bar{\mu }}{T}\;.$$

(see Figure 4). The same results are obtained from the thermodynamic derivative $S_{k}\left(T,\bar{\mu }\right)=-\partial K_{k}\left(T,\bar{\mu }\right)/\partial T$.

To conclude this section, elementary Fermi- and Bose-systems are characterized by *two* equations of state, one for the particle number $N_{\varepsilon }\left(T,\bar{\mu }\right)$ (usually termed the *distribution function*), the other for the entropy $S_{k}\left(T,\bar{\mu }\right)$. The first is ubiquitous in modern physics, while the latter is so far not much discussed in the present literature. $S_{k}$ describes in a concise form the caloric properties of these systems, which are the central building blocks of systems with indistinguishable particles. The reason for the lower prominence of $S_{k}$ is that the caloric properties of quantum gases are usually derived from the energy $E_{k}\left(T,\bar{\mu }\right)=\varepsilon \left(k\right)\cdot N_{k}\left(T,\bar{\mu }\right)$. Since ε(k) is not a variable, but a characteristic *constant* of the elementary subsystem labeled ‘*k*’, $E_{k}$ appears to be simply proportional to $N_{k}$, and hence not as an independent equation of state. This is different for the entropy $S_{k}\left(T,\bar{\mu }\right)$.

In usual terminology the elementary Fermi- and Bose-systems are called ‘single-particle states’, as they are related to the solutions of the one-particle Schödinger equation. The Hilbert space of the many-particle system is represented by the tensor product of the spaces of the single-particle systems. However, the requirement of exchange symmetry resulting from the indistinguishability of identical particles eventually prevents the use of single-particle systems (whose Hilbert space is spanned by the solutions of the one-particle Schrödinger equation) as independent building blocks of the many-body system. The exchange symmetry leads to quantum (Fermi- or Bose-like) correlations between the single-particle systems, destroying their statistical independence. These correlations are much more conveniently taken into account within the framework of second quantization used in Equation (36). The Schrödinger physics still enters in shape of the spatial distribution of the wave functions, which describe the spatial distribution of elementary Fermi- and Bose-systems. In other words, elementary Fermi- and Bose-systems are the excitation *modes* of the quantized matter field. These are statistically independent, and hence, the thermodynamic potential of the gas is just the sum of the thermodynamic potentials over all elementary subsystems. The particle numbers $\left\{N_{k}\right\}$ and the entropies $\left\{S_{k}\right\}$ of the elementary subsystems are independent random variables in the sense of thermodynamics, while the momenta of different particles are not independent, but subject to quantum correlations enforced by indistinguishability.

In the next step, we must assemble the elementary Fermi- and Bose-systems to quantum gases. To do this, we assume that the *k*-vectors compatible with the boundary conditions lie dense enough in k-space, and convert the sum over all elementary systems in into an integral over the energies [32]:

(43) $$\sum_{\vec{k}}\;=\;\frac{L^{d}}{{\left(2\pi \right)}^{d}}\int d^{d}k\;=\;L^{d}\int d\varepsilon \;g\left(\varepsilon \right)\;,$$

where g(ε) depends on the form of ε(k) and the systems’ dimensionality *d*; *L* being the spatial extension of the system in each direction. For spin 1/2 fermions, another factor of 2 must be added or spin has taken into account explicitly. In standard terminology g(ε) is called the density of (single-particle) states (DoS), from our point of view, it is the density of elementary Fermi- and Bose-systems on the energy axis.

Besides the Fermi- or Bose character, the *dispersion relation* ε(k) constitutes the main characteristic of the composite system, i.e., the specific quasiparticle gas under investigation. It is the only feature of our description that has a counterpart in classical physics [i.e., the Hamilton function $E(\vec{P})$]. The difference between the classical and the quantum point of view is the following: rather than saying that the same (classically distinguishable) particle is accelerated by external forces or a scattering process with another particle, the quantum point of view is that particles are *annihilated* in lower energy elementary Fermi- or Bose-systems, while a (possibly different) number of particles is *generated* in higher energy elementary systems (of course under the constraint of the conservation laws applicable to these transitions). The dynamical variables are not anymore the positions and momenta of individual particles (which are not relevant because of indistinguishability), but solely the particle number $N_{k}\left(T,\bar{\mu }\right)$, the entropy $S_{k}\left(T,\bar{\mu }\right)$, and the energy $E_{k}\left(T,\bar{\mu }\right)=\varepsilon \left(k\right)N_{k}\left(T,\bar{\mu }\right)$ of the elementary Fermi- and Bose-systems.

## 5. Application to Degenerate Quantum Gases

The explanation of the thermodynamic and transport properties of matter is the central goal of statistical physics. In particular, the thermodynamic derivatives entering the transport coefficients in the previous section, can be calculated using the methods of statistical thermodynamics. In the following we first derive the EoS for quantum gases and use the results as input to the drift-diffusion model to describe diffusive transport in metals and semiconductors.

Using the Fermi- and Bose distribution functions,

(44) $$N_{\varepsilon }\left(T,\bar{\mu }\right)\;=\;\frac{1}{\exp\left(\frac{\varepsilon -\bar{\mu }}{k_{B}T}\pm 1\right)}$$

it is straightforward to compute the thermal

(45) $$n\left(T,\bar{\mu }\right)\;=\;\int_{0}^{\infty }d\varepsilon \;g\left(\varepsilon \right)\cdot N_{\varepsilon }\left(T,\bar{\mu }\right)\;,$$

and the caloric

(46) $$e\left(T,\bar{\mu }\right)\;=\;\int_{0}^{\infty }d\varepsilon \;g\left(\varepsilon \right)\cdot N_{\varepsilon }\left(T,\bar{\mu }\right)\cdot \varepsilon$$

equation of state. The function g(ε) is called the density of states, and uniquely determined by the dispersion relation ε(k). From the above equations of state all the thermodynamic susceptibilities entering the transport coefficients of the drift-diffusion model (see Section 3) can be computed in equilibrium.

For degenerate electrons, e.g., in three dimensions, one obtains in effective mass approximation the DoS

$$g\left(\varepsilon_{F}\right)\;=\;\frac{3}{2}\frac{n}{\varepsilon_{F}\left(n\right)}\;\propto \;n^{1/3}\;,$$

with the Fermi energy

(47) $$\varepsilon_{F}\left(n\right)=\mu \left(T=0,n\right)=\frac{\hbar^{2}{\left(3\pi^{2}n\right)}^{2/3}}{2\hat{m}}\;,$$

where $\hat{m}$ is the mass per particle. One arrives at the well-known thermodynamic susceptibilities:

(48) $$\begin{array}{ccc} \nu & = & \frac{\partial n(T,\mu )}{\partial \mu }\;=\;g\left(\varepsilon_{F}\right) \end{array}$$

(49) $$\begin{array}{ccc} c_{v}\left(T,n\right) & = & \frac{\partial e(T,n)}{\partial T}\;=\;T\frac{\partial s(T,n)}{\partial T}=\frac{\pi^{2}}{2}\;\frac{nk_{B}^{2}T}{\varepsilon_{F}\left(n\right)}\;=\;s\left(T,n\right) \end{array}$$

(50) $$\begin{array}{ccc} \frac{\partial s(T,n)}{\partial n} & = & \frac{\pi^{2}}{6}\;\frac{nk_{B}^{2}T}{\varepsilon_{F}\left(n\right)}\;=\;\frac{\hat{s}\left(T,n\right)}{3}\;, \end{array}$$

where $\kappa_{T}$ the isothermal compressibility, and $c_{v}$ the thermal capacitance per volume at constant particle density.

On the other hand, for dilute gases, e.g., the electrons and holes in semiconductors, one obtains

(51) $$\begin{array}{ccc} \nu & = & \frac{\partial n(T,\mu )}{\partial \mu }\;=\;\frac{n}{k_{B}T} \end{array}$$

(52) $$\begin{array}{ccc} c_{v} & = & \frac{\partial e(T,n)}{\partial T}\;=\;T\frac{\partial s(T,n)}{\partial T}\;=\;\frac{3}{2}\;nk_{B} \end{array}$$

(53) $$\begin{array}{ccc} s(T,n)\; & = & \;\;nk_{B}\left\{\ln\left(\frac{jT^{3/2}}{n}\right)+\frac{5}{2}\right\}\;, \end{array}$$

(54) $$\begin{array}{ccc} \frac{\partial s(T,n)}{\partial n}\; & = & \;\hat{s}-k_{B}\;=\;k_{B}\left\{\ln\left(\frac{jT^{3/2}}{n}\right)+\frac{3}{2}\right\}, \end{array}$$

where $j=2\cdot {\left(\hat{m}k_{B}/2\pi \hbar^{2}\right)}^{3/2}$ may be called the *chemical constant* of the electron gas because it determines the absolute values of $\hat{s}$ and $\mu =\hat{e}-T\hat{s}+p\hat{v}$ (Equation (4)). Equation (53) is also known as the Sackur-Tetrode equation. Equation (54) provides a reasonable estimate for the thermopower of a doped (non-degenerate) semiconductor in the *T*-regime, where most dopants are ionized [33]. Because of the small factor $k_{B}T/\varepsilon_{F}$ in Equation (50) the thermopower of metals is usually much smaller than that of semiconductors.

The fact that the state of macroscopic system with $≃10^{23}$ internal degrees of freedom can be specified by only three independent variables (here *T*, *V*, and μ) is a consequence of the thermal and chemical equilibrium between the elementary subsystems. The composite character of macroscopic systems becomes visible, if the equilibrium between the subsystems is disturbed, e.g., by a laser, which selectively increases the population of elementary subsystems at higher energy at the expense of those at lower energies. The equilibrium is restored by inelastic recombination processes.

Are the elementary Fermi- and Bose-systems are fundamentally relevant as separate entities, or are just a technicality, which allows an easy computation of the EoS? This question is equivalent to the question of why the grand-canonical approach to statistical thermodynamics should be preferred to the micro-canonical and canonical ones. In equilibrium and in the thermodynamic limit, where the *k*-space can be considered to be a three-dimensional continuum, also the micro-canonical and the canonical approaches to thermodynamics work reasonably well. This changes when going beyond these restrictions as discussed in the next two sections. Historically, the characteristic statistical fluctuations of energy and particles numbers in these ensembles strongly biased the common view. Before the advent of quantum mechanics, fluctuations were considered possible only for open systems. Statistical fluctuations were a nuisance within classical physics, and their appearance within the canonical and grand-canonical ensemble was viewed as an artifact, which ought to be removed by the thermodynamic limit, i.e., the limit V,N→∞ at constant particle density *n*. From this perspective, the micro-canonical approach is often considered to be the most fundamental one, leading to the perception that thermodynamics as a whole works only within this limit. In quantum physics, however, statistical fluctuations constitute an unavoidable element of physics (see Section 10 for further discussion).

## 6. Ballistic Quantum Transport of Particles

In this section, it is shown that the elementary Fermi- and Bose-systems (which may look artificial at first sight) are very useful to understand transport properties in reduced dimensions, where the drift-diffusion model is entirely inapplicable. This is the regime of mesoscopic transport [34], where the size of the conducting object is smaller than the mean free path Λ. More precisely, it is even sufficient, if the *inelastic* mean free path $\Lambda_{in}\left(T\right)$, corresponding to those scattering processes that result in the dissipation of energy, is larger than the sample size.

Elastic scattering, i.e., scattering at constant energy, cannot create entropy because any increase ΔS of entropy requires the amount ΔE=TΔS of energy, which by definition of the term ‘elastic’ is not available. Instead, the elastic scattering modifies the underlying wave pattern, i.e., it changes the shape of the elementary Fermi- or Bose-systems in real space. The plane wave functions of ideal gases, or Bloch-wave functions of crystals, respectively, are then replaced by complex speckle patterns. Such speckle patterns are also known from diffraction of a laser beam by the dust on a dirty glass plate.

At low temperatures T≲1K the inelastic mean free path $\Lambda_{in}\left(T\right)$ is typically in the micron regime, while in high mobility semiconductor also the *elastic* mean free path easily exceeds the micron range, and enables the study of truly ballistic transport. In this regime it is possible to experimentally realize some simple textbook quantum systems, where plane waves are scattered off tunable potential barriers.

**Figure 5:**
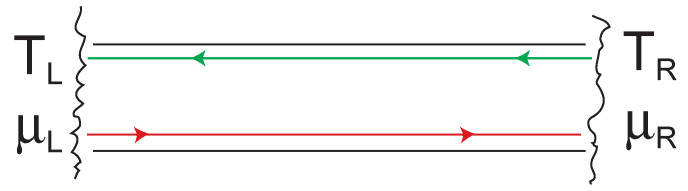
Schematic of single channel quantum wire connected to two reservoirs for energy, entropy, and particles. The quasi-one-dimensional character of the transport is ensured, once the transverse width of the wire is comparable to the Fermi wavelength. It can also be realized in wider strips via the formation of edge states in a quantizing magnetic field, i.e., in the quantum Hall regime. In this case, the magnetic field also provides a spatial separation between left- and right-movers. The elementary Fermi-or Bose-systems are charged with energy, entropy, and particles via the left (red) and the right (green) reservoir, respectively. They are in thermodynamic equilibrium with their source reservoirs, but not with each other.

The simplest case to consider is that of a quantum wire. To list a few examples, a quantum wire can be realized for photons (wave guides or optical fibers comparable with the wavelength of electromagnetic waves), for phonons (narrow suspended beams with a diameter comparable to the wavelength of thermally excited phonons), and for electrons (semiconductor heterostructures or carbon nanotubes with a diameter comparable to the Fermi wavelength). The case of an electronic quantum wire, of wave guide, is depicted in Figure 5. Such a wire essentially constitutes a narrow constriction between two particle reservoirs. Currents of particles, energy and entropy are driven by electrochemical potential and/or temperature differences between the reservoirs.

The common element in these examples of quantum wires is the fact that the set of allowed *k*-vectors does not form a three-dimensional continuum anymore, but consists of one, or a few one-dimensional sub-continua, which propagate particles, entropy, and energy along the wire (say, in *x*-direction), while the transverse part of the wave function is discrete, resulting from the strong confinement of the system in that direction. The one-dimensional sub-continua of elementary Fermi- or Bose-systems are also called *transport channels*. For simplicity, let us first assume that we have only one transport channel, as sketched in Figure 5. Initially, we also assume that the quantum wire is perfectly transmitting (T(ε)=1); smaller transmission coefficients T(ε)<1 are easy to take into account in the next step.

The transport of photons, phonons or electrons can then be viewed as a scattering problem: a beam of particles, emanating from two particle reservoirs connected to the left and right end of the wire, is either transmitted or reflected back. The elementary Fermi- or Bose-systems in the wire break up in two subsystems: *right-movers* and *left-movers*, which propagate particles, entropy, and energy with the dynamical velocity

(55) $$\vec{v}\left(k\right)=\frac{\partial \varepsilon (k)}{\hbar \;\partial k}\;,$$

and are populated according to the temperature and the electrochemical potential of the left and right reservoir, respectively [35]:

$$N_{\varepsilon ;\;L,R}\;=\;N_{\varepsilon }\left(T_{L,R},{\bar{\mu }}_{L,R}\right)\;.$$

In this approach, the elementary Fermi- and Bose-systems can be visualized as *conveyor belts* for energy, entropy, particles, momentum, and spin, which transport these quantities ballistically, until an elastic, or inelastic scattering event occurs.

If the two reservoirs differ in temperature or in (electro)chemical potential net currents of *E*, *S*, and *N* will flow. Despite being in a non-equilibrium state as a whole, the currents flowing through the wire of length *L* are perfectly described by the thermodynamic properties of the two (left- and right-moving) subsystems. Following Landauer and Büttiker [36,37,38,39] we can write for the particle current

(56) $$\begin{array}{ccc} I_{N}\; & = & \;\frac{1}{L}\left\{\sum_{k>0}N_{k,L}\;v\left(k\right)+\sum_{k<0}N_{k,R}\;v\left(k\right)\right\}\\ \;\\ & = & \;\int_{-\infty }^{\infty }d\varepsilon \;g\left(\varepsilon \right)\;v\left(\varepsilon \right)\;\left(N_{\varepsilon }\left(T_{L},{\bar{\mu }}_{L}\right)-N_{\varepsilon }\left(T_{R},{\bar{\mu }}_{R}\right)\right)\;, \end{array}$$

where $v\left(k\right)=-v\left(-k\right)$. In the second step we have evaluated the sum in a continuum approximation using the one-dimensional DoS $g\left(\varepsilon \right)=\left(1/\pi \right)\cdot dk_{x}\left(\varepsilon \right)/d\varepsilon$ for propagating modes in one dimension.

If we plug g(ε) into Equation (56) the energy dependence of the DoS and the velocity cancel, and we obtain the surprisingly universal result

(57) $$I_{N}\;=\;\frac{1}{\pi \hbar }\int_{-\infty }^{\infty }d\varepsilon \;\left(N_{\varepsilon }\left(T_{L},{\bar{\mu }}_{L}\right)-N_{\varepsilon }\left(T_{R},{\bar{\mu }}_{R}\right)\right)\;,$$

which is valid for both Fermi- and Bose-systems and independent of the functional form of the dispersion relation ε(k). In solid state nanostructures, phonon and photon currents are usually not detected by measuring electric current or counting particles, but as a thermal (i.e., ‘heat’) current. We leave the treatment of thermal currents to the next section and specialize now to charged systems, to compute the electric conductance and the thermopower of quantum wires.

If the wire hosts several transport channels with ε-dependent transparencies $T_{n}\left(\varepsilon \right)$, we must sum over all channels and the charge current $I_{Q}=\hat{q}\;I_{N}$ assumes the (with respect to Equation (56)) more general form

(58) $$I_{Q}\;=\;\frac{\hat{q}}{\pi \hbar }\sum_{n}\int_{-\infty }^{\infty }d\varepsilon \;T_{n}\left(\varepsilon \right)\;\left(N_{\varepsilon }\left(T_{L},{\bar{\mu }}_{L}\right)-N_{\varepsilon }\left(T_{R},{\bar{\mu }}_{R}\right)\right)\;.$$

To begin, we limit ourselves to the linear response regime and assume that the wire is symmetrically biased, i.e.,

(59) $$N_{\varepsilon ;\;L,R}=\;N_{\varepsilon }\left(T\pm \Delta T/2,\mu \pm \hat{q}U/2\right)\;,$$

where ΔT and $U=\left({\bar{\mu }}_{L}-{\bar{\mu }}_{R}\right)/\hat{q}$ are the applied temperature and voltage bias, and the upper and lower sign refer to the left and right reservoir, respectively. Then we can Taylor-expand $N_{\varepsilon ;\;L,R}$ of the reservoirs around the averages $(T_{L}+T_{R})/2$ and $({\bar{\mu }}_{L}+{\bar{\mu }}_{R})/2$ of *T* and $\bar{\mu }$, respectively. We obtain in linear approximation ($k_{B}\Delta T,\hat{q}U\ll k_{B}T$):

(60) $$\begin{array}{ccc} N_{\varepsilon }\left(T_{L},{\bar{\mu }}_{L}\right) & - & N_{\varepsilon }\left(T_{R},{\bar{\mu }}_{R}\right)\;=\frac{\partial N(Y)}{\partial Y}\;\Delta Y\\ & = & \;\frac{\partial N(Y)}{\partial Y}\underset{\Delta Y}{\underset{︸}{\frac{1}{k_{B}T}\left(\hat{q}U-\frac{\varepsilon -\mu }{T}\Delta T\right)}}\; \end{array}$$

where $Y=\left(\varepsilon -\mu \right)/k_{B}T$, and

(61) $$\frac{\partial N(Y)}{\partial Y}\;=\;\frac{\exp(Y)}{{\left(\exp(Y)\pm 1\right)}^{2}}\;.$$

The charge current then reads

(62) $$I_{Q}=\frac{\hat{q}}{\pi \hbar }\sum_{n}\int_{0}^{\infty }d\varepsilon \;T_{n}\left(\varepsilon \right)\;\frac{\partial N(\varepsilon )}{\partial \varepsilon }\;\left(\hat{q}U-\frac{\varepsilon -\mu }{T}\Delta T\right)\;.$$

In the case of fermions, a Sommerfeld expansion of the integral in Equation (62) leads to the relation

$$I_{Q}\;=\;G\;\cdot \;\left\{\;U\;+\;S\cdot \Delta T\;\right\}\;,$$

where in linear approximation

(63) $$G\;=\;G_{0}\cdot 2\sum_{n}T_{n}\left(\mu \right)\;,\;\mathrm{and}\;G_{0}\;=\;\frac{{\hat{q}}^{2}}{h}\;≃\;38.74\;\mu S\;≃\;\frac{1}{25.8\;k\Omega }\;.$$

The factor 2 takes into account spin degeneracy and $G_{0}$ denotes the universal *conductance quantum*. This is a seminal result of mesoscopic physics found experimentally first in gate-defined quantum point contacts [40,41]. This result holds in the linear regime, where an effect of the bias voltage on the set {T(ε)} can be neglected.

The thermoelectric counterpart of the conductance quantization, i.e., the Seebeck coefficient of a quantum wire, or quantum point contact is in first order given by

(64) $$S\;=\;\frac{\pi^{2}}{3}\frac{k_{B}^{2}}{\hat{q}h}\cdot 2\sum_{n}\;{\left.\frac{d\left(\ln T_{n}\left(\varepsilon \right)\right)}{d\varepsilon }\right|}_{\varepsilon =\mu }\;.$$

Additionally, this result has been experimentally confirmed first in quantum point contacts [42]. The energy current is computed analogously as

(65) $$\begin{array}{cc} I_{E}\; & =\;\frac{1}{\pi \hbar }\int_{-\infty }^{\infty }\;d\varepsilon \;\varepsilon \;\left[N_{\varepsilon }\left(T_{L},{\bar{\mu }}_{L}\right)-N_{\varepsilon }\left(T_{R},{\bar{\mu }}_{R}\right)\right]. \end{array}$$

## 7. Ballistic Quantum Transport of Entropy

The first efforts to transfer the ideas of ballistic electron transport to thermal transport originate from Imry and Sivan [43] and Butcher [44]. To describe the thermal transport through quantum wires, as illustrated in Figure 5, we can write down an expression for the entropy current that is the thermal analogue of Equation (39), but contains the entropy $S_{k;\;L,R}$ (Equation (41)) of the elementary Fermi- or Bose-systems rather than their particle numbers $N_{\varepsilon ;L,R}$. Assuming that the entropy propagates in each elementary Fermi-, or Bose-system at the same velocity $\vec{v}\left(k\right)$ as the particles, the entropy current reads:

(66) $$I_{S}\;=\;\frac{1}{\pi \hbar }\int_{-\infty }^{\infty }d\varepsilon \;\left(S_{\varepsilon }\left(T_{L},{\bar{\mu }}_{L}\right)-S_{\varepsilon }\left(T_{R},{\bar{\mu }}_{R}\right)\right)\;.$$

For the same symmetric bias (see Equation (59)) one finds in linear approximation

(67) $$\begin{array}{ccc} S_{\varepsilon }\left(T_{L},{\bar{\mu }}_{L}\right) & - & S_{\varepsilon }\left(T_{R},{\bar{\mu }}_{R}\right)\;=\frac{\partial S(Y)}{\partial Y}\;\Delta Y\\ & = & \;\frac{\partial S(Y)}{\partial Y}\frac{1}{k_{B}T}\left(\hat{q}U-\frac{\varepsilon -\mu }{T}\Delta T\right)\;, \end{array}$$

where again $Y=\left(\varepsilon -\mu \right)/k_{B}T$, and

(68) $$\frac{\partial S(Y)}{\partial Y}\;=\;k_{B}Y\cdot \frac{\exp(Y)}{{\left(\exp(Y)\pm 1\right)}^{2}}\;.$$

The upper sign holds for fermions, and the lower for bosons. Interestingly, this result differs from Equation (61) only by the extra factor $k_{B}Y$. For the entropy current we then find in linear response

(69) $$I_{S}=\frac{1}{\pi \hbar }\sum_{n}\int_{0}^{\infty }d\varepsilon \;T_{n}\left(\varepsilon \right)\;\frac{\varepsilon -\mu }{T}\frac{\partial N(\varepsilon )}{\partial \varepsilon }\;\left(\hat{q}U-\frac{\varepsilon -\mu }{T}\Delta T\right).$$

The first term ($\propto \Delta \bar{\mu }$) in this equation is driven by the voltage bias, and constitutes the ballistic analogue to the Peltier current in Equation (29). The second term (∝ΔT) describes the entropy current driven by the *T*-difference.

The same result is obtained, if one extends the derivation of $I_{N}$ to $I_{E}$ and computes $I_{S}$ via Equation (16):

(70) $$I_{S}\;=\;\frac{1}{T}\left(I_{E}-\bar{\mu }I_{N}\right)\;=\;\frac{1}{T}\left\{\Pi \cdot \;U\;+\;L\cdot \Delta T\right\}$$

where Π is the Peltier coefficient, and L being the thermal conductance. Please note that the identification of $I_{S}$ with $(I_{E}-\bar{\mu }I_{N})/T$ holds only in the linear response regime [45].

For fermions, we can again evaluate the integrals in Equation (69) within the Sommerfeld approximation, and obtain for the Peltier coefficient

$$\Pi \;=\;T\cdot \frac{2L_{0}}{\hat{q}}\;{\left.\sum_{n}\frac{d\left(\ln T_{n}\left(\varepsilon \right)\right)}{d\varepsilon }\right|}_{\varepsilon =\mu }\;,$$

and for the thermal conductance

$$L\;=\;T\cdot 2L_{0}\;\sum_{n}\left.T_{n}\left(\mu \right)\right.\;,$$

where the prefactors 2 accounts again for spin-degeneracy. The constant $L_{0}$ is the *entropy conductance quantum* corresponding to

(71) $$L_{0}\;=\;\frac{\pi^{2}}{3}\;\frac{k_{B}^{2}}{h}\;=\;0.9456\;{\mathrm{pW}/K}^{2}\;,$$

implying that *in ballistic quantum wires the entropy conductance L=L/T is quantized in units of $L_{0}$*. Compared to the quantum of electric conductance, ${\hat{q}}^{2}$ is replaced by ${\left(\pi k_{B}\right)}^{2}/3$. The rather universal ratio

(72) $$\begin{array}{c} L_{0}\;=\;\frac{L_{0}}{G_{0}}\;=\;\frac{\pi^{2}}{3}{\left(\frac{k_{B}}{\hat{q}}\right)}^{2}\;=\;24.4\;\mathrm{nW}\Omega /K^{2} \end{array}$$

of the two conductance quanta is called the Lorentz number, which governs the Wiedemann–Franz law: $L/G=T\cdot L_{0}$.

As discussed in the context of the drift-diffusion model in Section 3 the Seebeck- and Peltier-coefficients are connected by the Kelvin-Onsager relation

(73) Π=T·S.

In the present context, the Kelvin-relation results from the Maxwell-relation

(74) $$\frac{\partial N_{\varepsilon }\left(T,\bar{\mu }\right)}{\partial T}\;=\;\frac{\partial S_{\varepsilon }\left(T,\bar{\mu }\right)}{\partial \bar{\mu }}\;=\;-\frac{\exp(Y)}{T{\left(\exp(Y)\pm 1\right)}^{2}}\;,$$

between the derivatives of the two EoS (Equations (39) and (41)), and the fact that the transmission coefficients T(ε) determine all transport quantities [46].

These results are again very general, as they depend only on the energy dependence of the transmission coefficients, but neither on the dispersion relation nor on the particle statistics. After some more qualitative experiments [47] a quantitative experimental investigation of the thermal conductance of quantum point contacts has been performed only recently, exploiting the thermopower of quantum point contacts for local thermometry [48]. Very recently, also chiral thermal transport in the integer quantum Hall regime has been demonstrated [49].

In solid state physics, the most relevant cases for bosons deal with phonons and photons. In this case, we can set μ=0. The evaluation of the corresponding Bose-integral results in a quantized thermal conductance with the very same entropy conductance quantum $L_{0}$ as in the case of fermions [50,51,52]. The case of the quantized entropy conductance by phonons was first addressed by the beautiful experiments by Schwab et al. [53]. A few years later, Meschke et al. considered the case of microwave photons [54]. The latter case is of particular importance for instrumentation in mesoscopic physics. First, it explains why a careful filtering of the measurement leads at low temperatures is required: in a cryogenic setup the wires bring down energy and entropy not only via electronic and phononic thermal conduction, but also via thermal photons. These photons may not carry enough energy to heat up macroscopic objects such as the thermometer, but they can still induce jumps of charge carriers in electronic traps, or release single electrons from a quantum dot. Second, the photon case is interesting, because the techniques of microwave engineering provide possibilities to manipulate quantum photon thermal transport down to the mesoscopic scale [55].

## 8. Dissipation and Non-Linear Transport

So far, we have analyzed the transport Equations (57) and (66) in the linear response regime. In that regime, Equation (18) is well established [56] and is often used to compute the thermal current $TI_{S}$. Next, we ask, what happens to this relation at large bias in the non-linear regime, where entropy generation becomes important.

Let us first exploit the conservation laws for *N* and *E*: these allow us to connect the currents $I_{N}$ and $I_{E}$ with the temporal change of the enthalpy-like quantity

(75) $$\begin{array}{c} M\left(S,V,\bar{\mu }\right)\;:=\;E-\bar{\mu }N\;. \end{array}$$

To our knowledge, *M* has no generally accepted name; thus, we propose to call it the *grand-canonical enthalpy*, in analogy to the conventional enthalpy H=E+pV. The justification for such terminology is the fact that changes of *M* at constant *V* and $\bar{\mu }$ describe the energy transfer TΔS via the thermal channel:

(76) $$\begin{array}{c} dM\left(S,V,\bar{\mu }\right)\;:=\;dE-\bar{\mu }dN\;=\;\frac{\partial M(S,V,\bar{\mu })}{\partial S}\;dS\;=\;T\;dS\;, \end{array}$$

in precisely the same way, as changes of H(S,p,N) at constant *p* and *N* quantify the transfer of ‘heat’ in physical chemistry. If we apply this rule to our ballistic channel connecting a left and right reservoir, we find that the combined current $I_{E}-{\bar{\mu }}_{L,R}I_{N}$ is related to the rate of change of $M_{L,R}$ of the left and right *reservoir*. Thus, we arrive at the relations:

(77) $$\begin{array}{c} {\dot{M}}_{L,R}\;=\;\mp \left[I_{E}-{\bar{\mu }}_{L,R}I_{N}\right]\;, \end{array}$$

where the upper sign refers to the left reservoir. In other words, the entropy content of the two reservoirs changes with the rate:

(78) $$\begin{array}{c} {\dot{S}}_{L,R}\;=\;\mp \frac{1}{T_{L,R}}\left[I_{E}-{\bar{\mu }}_{L,R}I_{N}\right]\;. \end{array}$$

**Figure 6:**
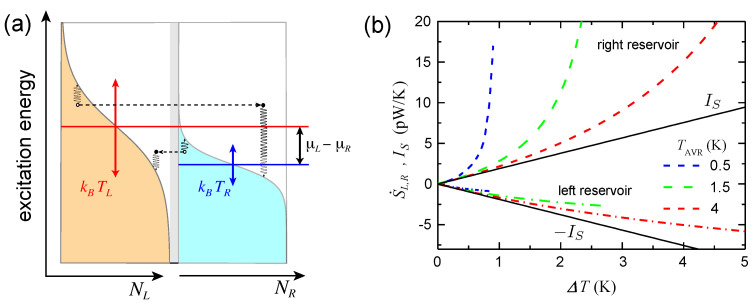
(**a**) Schematic of irreversible relaxation processes in the reservoirs following the transmission of a particle. Each transmission event causes inelastic scattering processes (wiggly lines) in *both* reservoirs that change the particle numbers of the elementary Fermi-systems until the reservoirs are in equilibrium again. (**b**) Colored dashed and dash-dotted lines: rates $S_{L,R}$ of entropy change in the left and right reservoir together with the entropy current $I_{S}$ (black solid lines) leaving the left and entering the right reservoir. The *T*-difference ΔT is varied, while the electrochemical potentials are kept equal. At very low ΔT the transport of entropy dominates over its production, while at larger ΔT, entropy production by the irreversible relaxation processes in the reservoirs governs their entropy change.

According to the continuity of entropy current $I_{S}$ (Equation (14) with X=S), the rates of entropy change in the reservoirs (with volumes $V_{L}$ and $V_{R}$)

(79) $$\begin{array}{c} {\dot{S}}_{L,R}\;\pm \;I_{S}\;=\;V_{L,R}\Sigma_{S;\;L,R} \end{array}$$

and the entropy current $I_{S}$ are not identical, but differ by the total entropy production rate $V_{L,R}\Sigma_{S;\;L,R}$. Please note that the *transport* through the elementary Fermi- and Bose-systems is *reversible*, provided that there is no inelastic scattering within the quantum wire—then all entropy production occurs exclusively in the reservoirs. Figure 6a illustrates the Fermi-functions in the reservoirs for the case that both *T* and $\bar{\mu }$ differ in the left and right reservoir. Transfer of a particle from left to right is accompanied by a corresponding entropy transfer given by Equation (41). The transfer leaves the reservoirs in a non-equilibrium state with an excess-electron in the right reservoir and an excess hole (equivalent to a missing electron) in one of the elementary Fermi-systems in the left reservoir. Restoring equilibrium Fermi-functions by electron–phonon scattering dissipates excess energy in both reservoirs.

For a perfectly transmitted channel (T=1), we can quantify these considerations, because the integrals in Equations (57), (65) and (66) can be performed in a closed form resulting in:

(80) $$\left.\begin{array}{cc} I_{N}\; & =\;\frac{k_{B}}{\pi \hbar }\left[T_{L}\phi_{0}\left(\frac{-{\bar{\mu }}_{L}}{k_{B}T_{L}}\right)-T_{R}\phi_{0}\left(\frac{-{\bar{\mu }}_{R}}{k_{B}T_{R}}\right)\right]\;,\\ I_{E}\; & =\;\frac{k_{B}}{\pi \hbar }\left\{k_{B}\left[T_{L}^{2}\phi_{1}\left(\frac{-{\bar{\mu }}_{L}}{k_{B}T_{L}}\right)-T_{R}^{2}\phi_{1}\left(\frac{-{\bar{\mu }}_{R}}{k_{B}T_{R}}\right)\right]+\left[T_{L}{\bar{\mu }}_{L}\phi_{0}\left(\frac{-{\bar{\mu }}_{L}}{k_{B}T_{L}}\right)-T_{R}{\bar{\mu }}_{R}\phi_{0}\left(\frac{-{\bar{\mu }}_{R}}{k_{B}T_{R}}\right)\right]\right\}\;,\\ I_{S}\; & =\;\frac{k_{B}^{2}}{\pi \hbar }\left\{T_{L}\left[\phi_{2}\left(\frac{-{\bar{\mu }}_{L}}{k_{B}T_{L}}\right)+\phi_{1}\left(\frac{-{\bar{\mu }}_{R}}{k_{B}T_{R}}\right)\right]-T_{R}\left[\phi_{2}\left(\frac{-{\bar{\mu }}_{L}}{k_{B}T_{L}}\right)+\phi_{1}\left(\frac{-{\bar{\mu }}_{R}}{k_{B}T_{R}}\right)\right]\right\}\;, \end{array}\;\right\}$$

where

$$\begin{array}{cc} \phi_{0}\left(x\right)\; & =\;-x+\ln(e^{x}+1)\;,\\ \phi_{1}\left(x\right)\; & =\;{\mathrm{Li}}_{2}\left(-e^{x}\right)+\frac{1}{6}\left(-3x^{2}+6x\ln\left(e^{x}+1\right)+\pi^{2}\right)\;,\\ \phi_{2}\left(x\right)\; & =\;{\mathrm{Li}}_{2}\left(-e^{x}\right)+\frac{1}{6}\left(-3x^{2}-6x\ln\left(e^{-x}+1\right)+6x\ln\left(e^{x}+1\right)+\pi^{2}\right)\;, \end{array}$$

and ${\mathrm{Li}}_{2}\left(z\right)$ being Spence’s function (dilogarithm). The entropy currents $I_{S}$ and the corresponding rates

$${\dot{S}}_{L,R}\;=\;\mp I_{S}+V_{L,R}\Sigma_{L,R}\;=\;\frac{{\dot{M}}_{L,R}}{T_{L,R}}\;=\;\mp \frac{I_{E}-{\bar{\mu }}_{L,R}I_{N}}{T_{L,R}}$$

of entropy change in the left and right reservoir are shown in Figure 6b for three different bath temperatures. The quantum point contact was subjected to a purely thermal bias (symmetrically around the average temperature), while the electrochemical potential was kept constant and equal in both reservoirs. Moreover, we assumed the reservoirs to consist of a two-dimensional electron gas in a GaAs quantum well with a fixed electrochemical potential of ${\bar{\mu }}_{L,R}=6$ meV. One sees that the ballistic entropy current varies nearly linear with temperature difference, while entropy production raises the rate of entropy change in both reservoirs in a non-linear fashion at higher ΔT. Analog consideration holds for the transmission of thermal phonons through suspended beams and thermal photons through optical wave guides and microwave transmission lines.

Experimentally, entropy flow cannot be detected directly. Only the reservoir observables, such as *T*, *S*, $\bar{\mu }$ and *N*, can be measured. Hence, at least in the non-linear regime, it is the ’heat flow’ $I_{E}-\bar{\mu }I_{N}$ and not $I_{S}$ that is experimentally accessible.

## 9. Connection to Diffusive Transport

**Figure 7:**
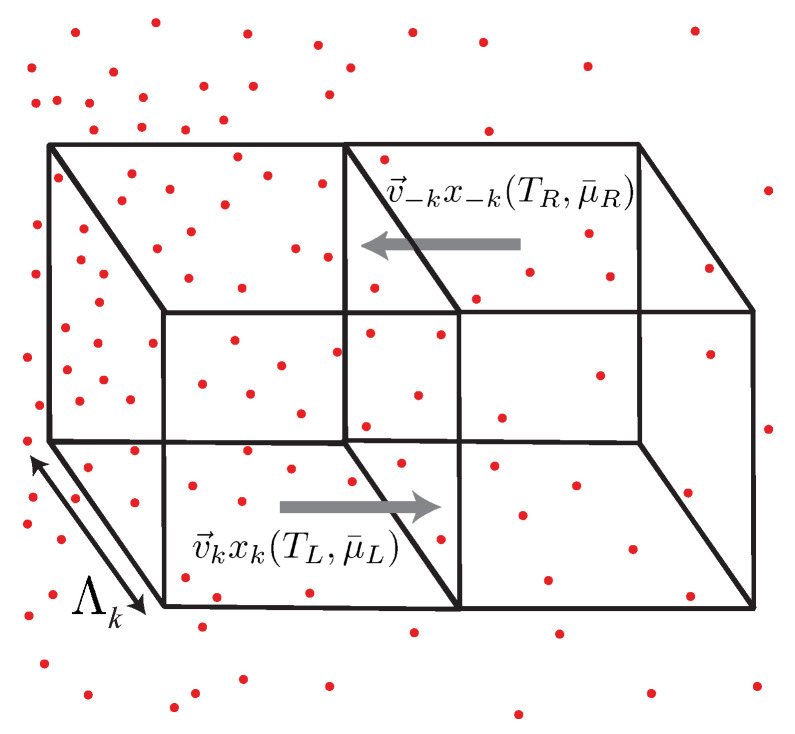
The left- (right-) propagating elementary Fermi- or Bose-systems labeled ‘$\vec{k}$’ emanating from the right (left) volume element of size $\Lambda_{k}$ contribute an amount $v_{k}x_{k}\left(T_{L,R},{\bar{\mu }}_{L,R}\right)$ to the total *X*-current density ${\vec{j}}_{X}$.

The concept of elementary Fermi- and Bose-systems can also be used to analyze the case of diffusive transport more accurately than in the simple drift-diffusion model. To do so, we consider similar to Section 3 two adjacent volume elements of linear dimension $\Lambda =|\vec{v}|\cdot \tau$, which are illustrated in Figure 7, and assume that an inelastic scattering mechanism exists, which establishes local thermal and electrochemical equilibrium on the scale of Λ. Volume elements of this size represent the smallest possible units, which can be assumed to be in local equilibrium. For a given *T*- or $\bar{\mu }$-gradient, two adjacent cubes of volume $\Lambda^{3}$ define the minimal distance, over which *T*- or $\bar{\mu }$-difference are physically sensible.

Between collisions, the elementary Fermi- and Bose-systems propagate energy, entropy and particles ballistically, as they do in the one-dimensional ballistic quantum wires discussed in the preceding sections. To account for the three spatial dimensions, we must average over the different *k*-directions, to determine the current density of any balancable quantity *X* in a given direction.

Similar to Section 3, we write the *X*-current density through the interface between the two cubes as the difference

(81) $${\vec{j}}_{X}\;=\;\sum_{k}\vec{v}\left(k\right)\cdot \left(x_{k}\left(T_{L},{\bar{\mu }}_{L}\right)-x_{k}\left(T_{R},{\bar{\mu }}_{R}\right)\right)\;$$

of left and right propagating currents, where $x_{k}$ is the contribution of each elementary subsystem to the total *x*-density depending on the values of $T_{L,R}$ and ${\bar{\mu }}_{L,R}$ within two adjacent cubes of size $\lambda_{k}^{3}$ (see Figure 7). The similarity of this expression to those used in ballistic transport is not accidental, but results from the fact that the propagation of any balancable quantity *X* is ballistic over distances smaller than the mean free path $\Lambda_{k}$. In the examples considered here $x_{k}$ is identified with the contribution $n_{k}=N_{k}\left(T,\bar{\mu }\right)/V$ or $s_{k}=S_{k}\left(T,\bar{\mu }\right)/V$ of an elementary Fermi- or Bose-system with the wave vector *k* to the particle density, or the entropy density, respectively.

In contrast to the more elementary treatment of the diffusive limit in Section 3 we now take into account that ${\vec{\Lambda }}_{k}=\vec{v}\left(\vec{k}\right)\cdot \tau_{k}$ depends on $\vec{k}$ via both $\tau_{k}$ and the velocity $\vec{v}\left(\vec{k}\right)$ of propagation between collisions. It is still a rather crude approximation, as it assumes that the scattering time is independent of the particle numbers in the other elementary Fermi- or Bose-systems. In linear approximation, the difference $\Delta x_{k}$ reads in analogy to the preceding Section 6 and Section 7

(82) $$x_{k,L}-x_{k,R}\;=\;\frac{\partial x_{k}\left(Y\right)}{\partial Y}\;\Delta Y\;,$$

where again $Y=\left(\varepsilon -\mu \right)/k_{B}T$, and ΔY is the variation of *Y* over the distance $\Lambda_{k}$ of ballistic propagation between scattering events. Similar to Equation (60) ΔY is given by

(83) $$\Delta Y_{k}\;=\;\frac{1}{k_{B}T}\;\left(\nabla \bar{\mu }-\frac{\varepsilon -\mu }{T}\;\nabla T\right)\cdot \underset{{\vec{\Lambda }}_{k}}{\underset{︸}{\vec{v}\left(k\right)\tau \left(k\right)}}\;.$$

Here, $|{\vec{\Lambda }}_{k}|$ is the mean free path associated with the scattering of quasiparticles in the elementary subsystem with wave vector *k*.

Plugging $\Delta Y_{k}$ into Equation (82) we obtain in the continuum limit

(84) $${\vec{j}}_{X}\;=\;\int_{0}^{\infty }d\varepsilon \;g\left(\varepsilon \right)D\left(\varepsilon \right)\frac{\partial x(\varepsilon )}{\partial \varepsilon }\left(\nabla \bar{\mu }-\frac{\varepsilon -\mu }{T}\;\nabla T\right)\;,$$

where the diffusivity tensor

(85) $$D\left(\varepsilon \right)=\;\int \int \;\frac{d\varphi }{2\pi }d\theta \sin\theta \;\vec{v}\left(\varepsilon ,\theta ,\varphi \right)⊗\vec{v}\left(\varepsilon ,\theta ,\varphi \right)\cdot \tau \left(\varepsilon ,\theta ,\varphi \right)$$

is the energy-dependent tensorial equivalent of the diffusion constant (averaged over the angles θ and φ on a sheet of constant ε(k)), which takes into account the shape and angle anisotropy of the dispersion relation ε(k) and the scattering time τ(k). For an isotropic ε(k) and τ(k) Equation (85) reduces to Equation (21).

Equation (84) constitutes the linearized transport equation for any balancable quantity *X*, and is in perfect agreement with the result from solutions of the Boltzmann equation in relaxation-time approximation in textbooks [56]. However, it is remarkable that its derivation here is based not on the classical concept of trajectories, but on the sole assumption of the existence of a scattering mechanism ensuring that the elementary Fermi- and Bose-systems propagate (on average) ballistically from opposite faces through a cube of size $\Lambda_{k}^{3}$ have values of $x_{k}\left(T,\bar{\mu }\right)$ according to the local values of $T(\vec{r})$, $\bar{\mu }\left(\vec{r}\right)$, and $T(\vec{r}+{\vec{\Lambda }}_{k})$, $\bar{\mu }\left(\vec{r}+{\vec{\Lambda }}_{k}\right)$, respectively.

The evaluation of Equation (84) for the particle and the entropy current reproduces the Drude formulas for the electric (Equation (24)) and the thermal (Equation (31)) conductivity, as well as the Wiedemann–Franz law. The evaluation of the thermopower and the Peltier coefficient within the Sommerfeld expansion leads to a slightly modified result, when comparing to the drift-diffusion model (see Equation (28)). Although the thermodynamic derivative ∂n(T,μ)/∂T entering Equation (28) involves only the derivative dg(ε)/dε (because the diffusion constant *D* is assumed to be independent of ε), the evaluation of Equation (84) contains the derivative $d\left(g(\varepsilon )D(\varepsilon )\right)/d\varepsilon$. The thermopower is then given by the Mott-formula

(86) $$S_{\mathrm{mott}}\;=\;\frac{\pi^{2}}{3}\frac{k_{B}^{2}T}{\hat{q}}\cdot \;{\left.\frac{d\left(\ln[g(\varepsilon )D(\varepsilon )]\right)}{d\varepsilon }\right|}_{\varepsilon =\mu }\;,$$

which is the diffusive analog of Equation (64). If both $g\left(\varepsilon \right)\propto \varepsilon^{\alpha }$ and $D\left(\varepsilon \right)\propto \varepsilon^{\beta }$ obey a power law, the logarithmic derivative in Equation (86) assumes the value

$${\left.\frac{d\left(\ln g(\varepsilon )D(\varepsilon )\right)}{d\varepsilon }\right|}_{\varepsilon =\mu }\;=\;\frac{\alpha +\beta }{\varepsilon_{F}}\;,$$

as opposed to the result $\alpha /\varepsilon_{F}$ obtained within the drift-diffusion model. For free electrons in three dimensions, we have α=β=1/2, if we assume the mean free path Λ to be independent of ε. In this simplest approximation, the result of Equation (86) is a factor of three larger than Equation (50), and we have the accidental relation

(87) $$S_{\mathrm{mott}}\;=\;\frac{\hat{s}\left(T,n\right)}{\hat{q}}\;.$$

This relation explains the observation that the low temperature molar entropy of charged Fermi-systems is so strongly correlated with the thermopower [22,57,58]. Of course, one should be aware that Equation (87) relies on the sometimes too simplistic relation α+β≃1. On the other hand, its experimental confirmation in Figure 3 works out pretty nicely, and can be considered to be a triumph of the quasiparticle picture in strongly correlated electron systems.

So far we have restricted our consideration to an isolated degenerate Fermi gas. A more accurate modelling must take into account that a solid usually contains several different Fermi- or Bose-gases. Besides the electrons, there are phonons, and (in magnetic solids) also magnons, or localized magnetic moments. The interaction between the different quasiparticle systems provides usually scattering mechanisms with an energy-dependent scattering time τ(ε), which may deviate from a power law, or at least, affect the value of β. In addition, there can be drag phenomena, such as electron–phonon, and electron–magnon drag, which can substantially complicate the behavior of S [59].

The mean free path $\Lambda_{k}$ (given by Equation (19)) can be calculated from the solution of the quantum-mechanical scattering problem of the relevant particles. It is quite intriguing that the model describes the *irreversible* process of conduction, without the need to explain how entropy is actually generated in the scattering process. As we will discuss in Section 10.4, the scattering cross section is calculated within standard Hamiltonian quantum theory, in which the time evolution is always reversible. Nevertheless, as long as the scattering enters only via a scattering *probability* (and not via a *probability amplitude*) in Equation (84), it is assumed that the phase memory in the scattering process is *erased*. This looks like a rather arbitrary truncation of the Hamiltonian time evolution that has some resemblance with the quantum-mechanical measurement process. It is this (here manually imposed) erasure of the phase memory, which gives rise to the ‘classical’ character of the Boltzmann-like transport theory. In the next section it is discussed, how phase coherence affects the transport. It turns out experimentally that it is the inelastic scattering processes, which account phenomenologically for the loss of phase coherence.

## 10. Discussion

### 10.1. Applicability of Thermodynamics

It is a widely spread opinion that the concepts of thermodynamics are applicable only in equilibrium, and for large systems with many microscopic degrees of freedom. The limit of large systems is also called the thermodynamic limit, where N,V→∞, and n=N/V=const. These restrictions result from the custom to *define* entropy via Planck’s famous formula

(88) $$S\;=\;k_{B}\;\ln\Omega \left(E,V,N\right)\;,$$

where Ω is the number of microstates accessible to *N* particles in a volume *V* with a given value of the total energy *E*. This strategy (when properly combined with the principle of indistinguishability) indeed allows the calculation of the function S(E,V,N), which is equivalent to E(S,V,N), and hence constitutes a starting point for equilibrium thermodynamics in entropy representation. However, in so-called *open* systems, which exchange energy and particles with the environment, the number Ω of microstates cannot be defined anymore, and hence it seems that thermodynamics is inapplicable to transport situations.

On the other hand, the presentation of the preceding sections shows that thermodynamics *can* well be applied to a wide variety of non-equilibrium and transport situations, *provided that a proper decomposition into simpler subsystems is chosen*. To be more specific, in a ballistic quantum wire such as the one-dimensional subbands formed in high mobility two-dimensional electron systems (see Figure 5), the set of right- (left-)moving elementary Fermi, or Bose-systems are in equilibrium with each other and the left (right) reservoir, but the equilibrium between left and right-movers is disturbed by the applied bias voltage, or the temperature difference, respectively. The same holds locally in a macroscopic piece of matter subjected to a *T*- or $\bar{\mu }$-gradient: also here left- and right moving elementary Fermi- and Bose-systems are out of equilibrium, but the amount of entropy and particles stored in the elementary Fermi- or Bose-systems *propagating in the same direction from a certain point in space* is to a very good approximation given by the local values of the temperature and (electro)-chemical potential.

This explains, why the current densities of entropy and particles are determined by the diffusion coefficient and the local (equilibrium) values of the derivatives of particle and entropy $[n_{k}\left(T,\bar{\mu }\right)$ and $s_{k}\left(T,\bar{\mu }\right)]$ density, respectively.

In this sense the thermodynamic concepts retain their relevance, irrespective of a reduced dimensionality or the absence of global equilibrium. The robustness against global non-equilibrium is also intuitively clear, as we do not question the validity of the concept of water temperature based on the undisputable absence of thermal equilibrium between the Mediterranean and the Polar Sea. That the same concepts remain valid in quantum wires with perfect transmission down to the atomic scale, e.g., in highly transparent atomic point contacts, appears more surprising, but has been demonstrated in a variety of beautiful experiments [60,61,62].

It must be pointed out that a large body of literature considers the Hamiltonian dynamics of small quantum systems coupled to reservoirs, striving for a modelling of nanoscale thermodynamic processes (see, e.g., Ref. [63] and the references therein), including the transfer of work and heat between nanosystems and the reservoirs. In contrast the present review is focused on simple transport phenomena, and tries to elucidate the conceptual foundation, on which the standard approach to both ballistic and diffusive transport is so successful.

### 10.2. Breakdown of Local Equilibrium

**Figure 8:**
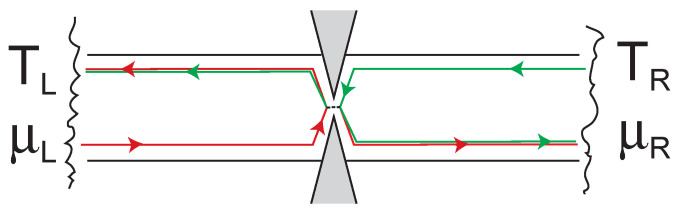
The same quantum wire as in Figure 5, but interrupted by a quantum point contact (QPC) with transmission coefficient T(ε). The scattering states of the particles emanating from the left (red) and the right (green) reservoir, respectively, must be considered to be *one* elementary Fermi- or Bose-systems, which cannot be further decomposed into subsystems.

The Landauer-Büttiker approach to transport restricts itself to the particular case of a nanoscale constriction between macroscopic reservoirs, in which the *thermodynamics* of the elementary Fermi- or Bose-systems in the constriction is governed by the reservoirs, and can be separated from the *transmission properties* of the constriction expressed by the set $\left\{T_{n}\left(\varepsilon \right)\right\}$ of transmission coefficients [64]. This separation holds best in the linear regime, and as long as energy and particle number of the elementary subsystems differing in the characteristic energy ε are statistically independent, because no inelastic scattering induces an exchange of energy, entropy and particles between them. In this way, the population of the reservoirs, which are by definition in internal equilibrium, is transferred to the elementary subsystems of the quantum wire. These can be considered to be in thermodynamic equilibrium with their respective source reservoir, while there is no equilibrium between elementary subsystems charged by *different* reservoirs, such as the left- and right-movers in Figure 5 and Figure 8.

Under these conditions, the propagation of energy, entropy and quasiparticles within the constriction is governed by reversible Hamiltonian dynamics, while the conceptionally difficult irreversible equilibration of the injected quasiparticles within the reservoirs *is irrelevant for the transport properties of the constriction*! This explains the success of the Hamiltonian dynamics in the description of this category of transport processes. It is interesting that this success can (within the semi-classical approximation) also be transferred to the macroscopic case, where reservoirs are absent, and a sufficiently strong inelastic scattering ensures local thermodynamic equilibrium, if one averages over volumes larger than the mean free path (see Section 9).

If elastic scattering occurs within the phase coherent region, the elementary subsystems labeled {k} in the absence of scattering must be replaced by more complicated ones, which in usual terminology are called *scattering states*, and consist of the incoming and the two (or more) outgoing waves. Such a complex wave pattern still forms *one* elementary Fermi- or Bose-system that cannot be decomposed further. In particular, it is impossible to provide a local thermodynamic description of the quantum wires left and right of the quantum point contact in Figure 8. The quantum point contact partitions the flux of energy, entropy and quasiparticles emanating from each reservoir according to the transmission coefficient T(ε) into the two corresponding outgoing fluxes. As the particles emanating from different reservoirs are *incoherent*, and thus statistically independent, their average particle numbers in the elementary Fermi-and Bose-systems flowing into the right reservoir simply add up according to

(89) $$N_{k}^{\mathrm{neq}}\;=\;T\left(\varepsilon \right)\cdot N_{k}^{\mathrm{eq}}\left(T_{L},{\bar{\mu }}_{L}\right)+\left(1-T\left(\varepsilon \right)\right)\cdot N_{-k}^{\mathrm{eq}}\left(T_{R},{\bar{\mu }}_{R}\right)\;,$$

while we have for particles entering the left reservoir

(90) $$N_{-k}^{\mathrm{neq}}\;\;=\;\left(1-T\left(\varepsilon \right)\right)\cdot N_{k}^{\mathrm{eq}}\left(T_{L},{\bar{\mu }}_{L}\right)+T\left(\varepsilon \right)\cdot N_{-k}^{\mathrm{eq}}\left(T_{R},{\bar{\mu }}_{R}\right)\;.$$

Here $N_{k}^{\mathrm{eq}}$ denotes the equilibrium particle numbers in the reservoirs. According to Equation (57), the non-equilibrium particle numbers $N_{k}^{\mathrm{neq}}$ determine the current. Analogous expressions hold for $S_{k}$, $E_{k}$, and all other balancable quantities of the system.

As it is a superposition of equilibrium particle numbers, and entropies with different $\left\{T,\bar{\mu }\right\}$, the particle numbers $N_{k}$ in the outgoing branches do not satisfy Equation (39) anymore. This signals the breakdown of local equilibrium. Similarly, the entropies $T\left(\varepsilon \right)S_{k}$ and $\left(1-T(\varepsilon )\right)S_{k}$ propagated by the outgoing partial waves after the scatterer are *lower* than the entropy of an equilibrium mode with the same temperature of the left reservoir (see Equation (41)) and particle numbers $T\left(\varepsilon \right)N_{k}\left(T,\bar{\mu }\right)$ and $\left(1-T(\varepsilon )\right)N_{k}\left(T,\bar{\mu }\right)$, respectively.

One may be tempted to consider a single quantum wire between the scattering region and one reservoir as a ‘system’ on its own, and ask for its thermodynamic quantities, e.g., its particle number or entropy. The wire segment leading to the QPC, however, is certainly not an independent subsystem in the sense of thermodynamics, because its particle numbers and other physical quantities are correlated with those of the other quantum wires connected to the constriction. The correlation of the particle numbers can be experimentally accessed, e.g., as an anti-correlation between the particle currents in the two reservoirs [65]. Thus, it must be kept in mind that the transport modes, i.e., the set of elementary Fermi- and Bose-systems hosted by the scattering region and the quantum wires cannot be decomposed in smaller subsystems as long as their phase coherence is not destroyed by inelastic processes.

A particularly nice experimental demonstration of such out-of-equilibrium physics was recently obtained using the one-dimensional edge channels in the quantum Hall regime [66,67]. There it was possible to measure the ε-dependence of the particle numbers $N_{\varepsilon }$ at different distances (0.8–30 µm) from a quantum point contact. Close to the quantum point contact a double step shape of $N_{\varepsilon }$ was observed, which resulted from the superposition of the transmitted and reflected electrons emanating from of reservoirs at different electrochemical potential (see Equations (89) and (90). At larger distances inelastic scattering processes restored the local thermal and chemical equilibrium, and $N_{\varepsilon }$ behaved Fermi-like. The measured local temperature was higher than that in the reservoirs, but lower than expected from energy conservation arguments. One possibility to explain the apparent loss of energy are additional *neutral modes*, which are predicted to exist, when two (spin-degenerated) edge channels are present [68].

A breakdown of local equilibrium can also happen in the diffusive regime. As shown in a series of experiments on diffusive wires of length *L* in the μm-regime this is possible at low temperatures, if the average diffusion time $\tau_{D}=L^{2}/D$ through the wire is shorter than the time required for energy relaxation in the wire [69]. This means the energy relaxation occurs predominantly by inelastic scattering in the two macroscopic reservoirs serving as contacts for the wire. If the energy relaxation in the wire is entirely negligible, no particle exchange occurs between elementary Fermi-systems of different characteristic energy ε—i.e., elastic scattering processes by static impurities dominate in the wire. The effect of the elastic scattering is an efficient randomization [70] of the momentum distribution in the wire, which corresponds to intense exchange of particles (and entropy) between elementary Fermi-systems with the same ε, but differing in *k*-direction [71]. In absence of inelastic scattering the elementary Fermi-systems with the same ε are not in local equilibrium, because $N_{\varepsilon }$ and $S_{\varepsilon }$ at any point in the wire have contributions from both reservoirs.

To quantify these considerations, it is simplest to consider the diffusion equation for the particle density $n_{\varepsilon }\left(r\right)$ in elementary Fermi-systems with the same ε (*r* is the position along the wire) with a source term $\Sigma_{N_{\varepsilon }}$ describing the exchange of particles with elementary Fermi-systems with other ε. The diffusion equation results from the combination of the continuity equation Equation (14) with Fick’s law (Equation (22)) for the particle densities $n_{\varepsilon }\left(r\right)$ in the composite subsystem containing all elementary Fermi-systems with the same characteristic energy ε:

(91) $$\frac{\partial n_{\varepsilon }\left(\vec{r},t\right)}{\partial t}+D\left(\varepsilon \right)\;\mathrm{divgrad}\;n_{\varepsilon }\left(\vec{r},t\right)\;=\;\Sigma_{N_{\varepsilon }}\;.$$

The source term $\Sigma_{N_{\varepsilon }}$ is called the *scattering integral*, and consists of a sum over all possible transitions between one elementary Fermi-system and the others with the quantum-mechanical transition probabilities $W_{\varepsilon ,\varepsilon^{\prime }}$ [69]. For isotropic scattering only the energy dependence of the $W_{\varepsilon ,\varepsilon^{\prime }}$ is relevant, and $\Sigma_{N_{\varepsilon }}$ reads

(92) $$\Sigma_{N_{\varepsilon }}\;=\;\int d\varepsilon^{\prime }\;g\left(\varepsilon \right)g\left(\varepsilon^{\prime }\right)\;\times \left\{W_{\varepsilon ,\varepsilon^{\prime }}n_{\varepsilon }\left(1-n_{\varepsilon^{\prime }}\right)-W_{\varepsilon^{\prime },\varepsilon }n_{\varepsilon^{\prime }}\left(1-n_{\varepsilon }\right)\right\}\;.$$

In this case, a relaxation-time approximation is not sufficient to describe the experimental data, which are accurate enough to extract the energy dependence of the scattering probabilities $W_{\varepsilon ,\varepsilon^{\prime }}$.

If inelastic scattering occurs only in the reservoirs, but is negligible in the wire, the particle generation and annihilation term $\Sigma_{N_{\varepsilon }}$ can be neglected in Equation (91) and the resulting $n_{\varepsilon }$ is a two-step function composed of the two Fermi-functions of the reservoirs with weight factors r/L and 1−r/L and that vary with the position *r* along the wire between 0 and 1 (*L* is the length of the wire). If the inelastic scattering among the electrons is very strong, but negligible between electrons and phonons (relevant for short wires), local thermal equilibrium is re-established and $n_{\varepsilon }$ is a Fermi function with a spatially varying electron temperature $T_{\mathrm{el}}\left(r\right)$. The resulting temperature profile can be determined by solving the thermal diffusion equation with a source term resulting from the local energy dissipation [69]. For very long, narrow wires the profile of $T_{\mathrm{el}}\left(r\right)$ at sufficiently low temperatures becomes flat, but remains elevated with respect to the phonon temperature, since the electron–phonon coupling becomes very weak in this limit [72].

### 10.3. Interference of Elementary Fermi- and Bose-Systems

So far, we have not taken into account quantum interference between the different modes, or elementary Fermi- and Bose-systems. The effect of interference is simply that two or more modes are not independent anymore, but form *new* modes, i.e., the coherent superpositions of the interfering modes. These new elementary subsystems can be charged with energy, entropy and particles only as one entity, with a transmission coefficient T(ε) telling us how constructive or destructive the interference is. The simplest example for such a coherent superposition is the standing waves, which are formed in a quantum well, or a finite quantum wire, by multiple reflection at the confining potential walls. In this case, the new systems cannot support a stationary current. Finite currents can be carried only by propagating waves, i.e., scattering ‘states’, provided that the phases of the interfering waves are adjusted such that the transmission probability is finite. For a completely destructive interference again a standing wave is formed, which suppresses the transport of entropy and particles, and the transport currents impinging on the interferometer are reflected completely. If the source that charges the propagating modes is at a finite temperature, the local current densities of energy, entropy and particles are invariably connected.

The elastic scattering does not produce entropy, but results in a *coherent* branching of the flows of energy, entropy and particle currents by the scatterer. This statement remains valid for arbitrary complex scattering regions and any number of terminals. The phase coherence can be made visible, if the partial waves are brought to interference, e.g., by adding a second scatterer or semi-transparent mirror, resulting in an electronic or photonic interferometer of the Fabry-Perot [73], Michelson- or Mach-Zehnder type [74,75,76].

For these reasons, the possibility of phase-tuning of the transmission coefficients $T_{k}$, and a corresponding phase-dependent thermal conductance [77,78] not very surprising. The close correspondence between the transport of particles and entropy resulting from the present approach renders entropy- or ‘heat’-interferometer [79] as natural as a quasiparticle-interferometer, even though the entropy stored within one elementary Fermi- or Bose-system results from an *incoherent* superposition of states with different particle numbers. The stored entropy leaves coherent superposition of the elementary Fermi- or Bose-system with others unaffected, as the superposition affects only the transmission coefficients, but not the $N_{k}\left(T,\bar{\mu }\right)$ and $S_{k}\left(T,\bar{\mu }\right)$ responsible for their thermodynamic properties.

Quantum interference of particles [80,81] and entropy [82] also occurs in diffusive systems. In this case, the modes of the system cannot be chosen as plane waves anymore, but constitute complex scattering states formed by the interference of waves on multiple scattering centers. The dominating interference contribution to the transport comes from pairs of time reversed diffusion paths, which return to the starting points. The pairs of time reversed paths interfere destructively (at zero external field). The interference increases the probability of return to a given point ($W_{\mathrm{return}}=1/\sqrt{4\pi Dt}$ for diffusion in one dimension) by a factor of 2, and thus results in a reduction of the conductivity. Here, the diffusion constant takes into account elastic scattering processes only. The inelastic scattering time $\tau_{\mathrm{in}}$ determines the typical length $L_{\mathrm{in}}=\sqrt{D\tau_{\mathrm{in}}}$ of phase coherent diffusion paths. This effect, known as weak localization, can be taken into account as an interference contribution to the diffusion constant *D* with respect to Equation (21). Coherent backscattering of light has also been observed. An external magnetic field tunes the character of the interference between the different diffusion paths in a continuous way between constructive and destructive (Aharonov-Bohm effect). The total transmission probability of a set of interfering modes strongly depends on the wavelength (i.e., on ε) and via the Aharonov-Bohm effect on the magnetic field *B*—leading to characteristic conductance fluctuations when $\varepsilon_{F}$ or *B* are varied.

In conclusion, the concept of elementary Fermi- and Bose-systems turns out to be extremely flexible. It can be adapted to a wide range of applications in modern physics. Once accepted, it provides a more reliable guide for our intuition than the classical concept of moving particles, as it incorporates the non-classical concepts of quantum interference and indistinguishability from the start.

### 10.4. Irreversibility and the Loss of Phase Coherence

The most ingenious side of the Boltzmann equation is the fact that the aspect of irreversibility, i.e., the generation of entropy is incorporated in the *ad-hoc* assumption of the existence of a scattering mechanism, and the corresponding characteristic length $\Lambda_{\mathrm{in}}$. As noted very early, such a mechanism is inconsistent with the notion of Hamiltonian dynamics. Any system with a discrete energy spectrum is subjected to the *recurrence objection*, i.e., its time evolution must be reversible. The recurrence objection is removed by assuming the existence of an infinite thermal bath with a continuous spectrum, in which energy and entropy can be dumped without recurrence. Such an approach is successful for systems with a single or a few macroscopic degree of freedom such as quantum bits, coupled to many microscopic degrees of freedom. Irreversibility is then generated by ‘tracing out’ the bath degrees of freedom.

Phenomenologically, the generation of entropy can be accounted for by damping out the off-diagonal elements of the density matrix, which are responsible for the coherent Hamiltonian dynamics. In the limit of long times, the decoherence becomes complete, implying a density matrix, which is diagonal in the basis of energy eigenstates, and with the probabilities $\left\{W_{i}\right\}$ as eigenvalues. The joint dynamics of the system and the bath features thermally induced temporal fluctuations of physical quantities of the system. These fluctuations obey the recently much discussed *fluctuation theorems* [83].

A first principles derivation of irreversibility remains a severe problem, as the ‘first principles’ at hand are all reversible. In the opinion of the author, it is not clear, whether the mathematical operation of ‘tracing out the bath degrees of freedom’ has some correspondence on the experimental side. Moreover, there are situations such as the collisions of heavy ions at very high energy, where vast amount of entropy is generated on such short time scales (1 fm/$c\approx 10^{-23}\;$s) that no sufficiently strongly coupled bath may exist. An example are events are known as ‘little bangs’, as opposed to the big bang of cosmology (see, e.g., [84]).

## 11. Conclusions

The purpose of this paper is to provide a coherent and self-contained description of the transport of particles and entropy both in the macroscopic and the mesoscopic regime. To implement this program, the concepts of thermodynamics first must be formulated in a way that avoids unnecessary limitations. To connect the general principles of thermodynamics to the quantum physics of matter, the idea of *ballistically moving particles* or *plane wave propagation*, respectively, must be stripped from all classical elements. It is proposed to use the *eigenmodes of the matter field* in the language of 2nd quantization as the elementary building blocks of such a description. Viewed as thermodynamic systems, called here *elementary Fermi- and Bose-systems*, they can be taken as a basis for a unified description of *both* global thermodynamic equilibrium *and* the ballistic and diffusive quantum transport. In this description ’classical’ and quantum transport rely on the very same principles, while the only demarcation line runs between regimes, where dissipation, i.e., the loss of phase coherence, occurs locally, or remotely in macroscopic reservoirs. The notion of elementary Fermi- and Bose-systems may prove useful not only in solid state physics, including the presently unfolding fields of spintronics, caloritronics, and spin-caloritronics, but also in the description of ultracold atomic and molecular gases [85].
